# Analysis of qualitative and quantitative morphological traits related to yield in country bean (*Lablab purpureus* L. sweet) genotypes

**DOI:** 10.1016/j.heliyon.2022.e11631

**Published:** 2022-11-22

**Authors:** Rahima Khatun, Md. Imtiaz Uddin, Mohammad Mahir Uddin, Mohammad Tofazzal Hossain Howlader, Muhammad Shahidul Haque

**Affiliations:** a Department of Agriculture Extension (DAE), Government Of the People’s Republic of Bangladesh and Department of Biotechnology, Bangladesh Agricultural University, Mymensingh 2202, Bangladesh; b Biotechnology Division, Bangladesh Institute of Nuclear Agriculture, Mymensingh 2202, Bangladesh; c Department of Entomology, Bangladesh Agricultural University, Mymensingh 2202, Bangladesh; d Insect Biotechnology and Biopesticide Laboratory, Department of Entomology, Bangladesh Agricultural University, Mymensingh 2202, Bangladesh; e Department of Biotechnology, Bangladesh Agricultural University, Mymensingh 2202, Bangladesh

**Keywords:** Lablab bean, Germplasm, Morphological traits, Multivariate, Characterization

## Abstract

Country bean is a grain legume extensively farmed for its multi-purpose uses, yet the traits related to yield are are poorly studied and yet unexplored. A study on the diversity of qualitative and quantitative morphological characteristics concerning yield among the country bean germplasms collected from Bangladesh identified considerable variation in the studied traits across the germplasms and identified a complex correlation between the qualitative and quantitative traits. Principal Component Analysis (PCA) detected five components that contributed 66.38% qualitative traits and six components contributed 74.49% quantitative traits to total variations. Eigenvalues indicated that a majority of color-related qualitative traits included cotyledon, leaf, vein, seed, flower, and petals contributed, in contrast,a majority of the seed, leaf, flower, and inflorescence-related quantitative traits contributed to the total diversity of the *Lablab* germplasms. Among the quantitative traits, the highest coefficient of variation (CV%) was found in average pod weight (50.98%), followed by the total number of spikes per plant (43.82%), while seed length, pod weight, length, width, thickness, number of flower/spike, spike length, and total no of spikes/plant all had more than 20.00 percent CV, suggesting suitability to use in the breeding of high yielding genotypes. The germplasms are grouped into four and three clusters based on quantitative and qualitative traits, suggesting quantitative characters offer better clustering of genotypes. Considering the above traits, our research found that the BD-10804, BD-10807, BD-11091, BD-10808, BD-10815, and BD-11089 and cultivar Goal Goda *Lablab* beans germplasms produced higher pod weight with corresponding higher pod length, width, and thickness suggesting to use them as high yielding genotypes for food and fodder purposes.

## 1 Introduction

Country bean, *Lablab purpureus* (L.) Sweet (Fabaceae) is one of the oldest cultivated plants, with records dating back to 1500 BC in India (Fuller, 2003) and the 4th century AD in Egyptian Nubia (Clapham and Rowley-Conwy, 2007). The plant is presently grown throughout tropical regions in Asia and Africa either as mono-crop or mixed crops. It is one of the most diversified legume crops domesticated to use as pulse, vegetable (green bean, pod, and leaf part), green manure, and ornamental crops (Maass et al., 2010; NRC, 2006). The crop also appears to be used for medicinal use in pharmaceutical or nutraceutical sectors (Morris, 2009). The young and immature green pods are cooked as vegetables. The pods are rich in proteins (22.4–31.3%) and carbohydrates (55%) and can be a perfect substitute for expensive animal proteins. The pods also contain vitamins and minerals such as copper, potassium, magnesium, iron, and phosphorus, are fiber-rich and are suitable for human nutrition (Adnan et al., 2021).

Country bean is one of the most popular vegetables in Bangladesh. This crop is grown in all 64 districts and almost every village in the homestead garden in Bangladesh. Although beans are cultivated in the winter, they become a year-round crop as photo-insensitive, and summer varieties become available (Nisha et al., 2021; Ahmed *et el*., 2004; Biswas 2015). In Bangladesh, growers produced about 1,69,735 metric tons of country beans from 61,628 acres (BBS, 2020). The yield potential of country bean varieties is comparatively low, and the traits contributing to yield are poorly studied and thus remain unexploited.

Bangladesh is an important geographical location with rich biodiversity for plants, animals, etc. There are enormous variations in the plant, seed, flower, and pod characters among the germplasms grown in Bangladesh. The success of any general and specific trait improvement breeding program largely depends on the diversity and variation present among the germplasms. Enhancing yield and resistance to biotic and abiotic stresses are significant thrusts for crop improvement programs. A considerable number of native strains of lablab bean are available in Bangladesh, and the researchers of the Plant Genetic Resources Centre (PGRC), Bangladesh Agricultural Research Institute (BARI), Bangladesh, have collected 551 germplasm of lablab bean from different locations in Bangladesh (Islam, 2008). Few previous studies were made to characterize some germplasm (Islam et al., 2002; Islam 2004).

Few studies have explored the diversity of lablab bean germplasms qualitative and quantitative morphological traits related to yield. Eighty-eight lablab bean germplasms from 14 districts of Bangladesh were assessed using fifteen characters, and considerable variation was reported (Islam, 2008). Major morphological characters of 117 lablab bean germplasm samples, 20 of which were from Bangladesh, and the rest from 20 different countries, have been evaluated previously (Sultana et al., 2001). Although there were variations across the germplasm, no distinctive regional gene pool was found. The morphology of wild-type accessions was highly similar, whereas Asian accessions had a lot of variances (Sultana et al., 2001). A relatively wide range of variations (21–36%) was observed among qualitative traits such as the color of hypocotyl, epicotyl, stem, node, leaf, and leaf vein among lablab bean germplasms reported collected from Bangladesh (Islam et al., 2009). However, a systematic study using the qualitative and quantitative morphological diversity of lablab beans is rare.

Multivariate analysis approaches may be help determine genetic diversity and in categorizing germplasms (Uyeda et al., 2015; Badenes et al., 2000). Among these techniques, Principal Component Analysis (PCA) is a statistical approach for categorizing many variables into primary uncorrelated variables. PCA can be employed to identify germplasm characterization features, visualize individuals' differences and relationships, and assess their contribution to total variation (Singh et al., 2016; Martínez-Calvo et al., 2008). Furthermore, hierarchical cluster analysis may be utilized to categorize and organize germplasm (Manjunatha et al., 2007; Aghaei et al., 2008; Karimi et al., 2009).

Molecular marker approaches for genotype description have also been effective, although they are costly and need to be identified with marker-linked trait data (Hasan et al., 2021; Holland, 2004). In contrast, the characterization of different morphological traits is the first step to describing and classifying germplasms of various crops (Balduzzi et al., 2017; Jingura and Kamusoko, 2015). To choose parents and their progenies, a document of morphological features is necessary for characterizing and categorizing germplasms. Understanding the phenotypic variability provided by various morphological and agronomical characteristics, such as seed, leaf, plant, and fruit-related characteristics is crucial for the conservation, breeding strategies, development, and commercialization of new varieties (Gonçalves et al., 2008) having high yield. Choosing appropriate morphological features is also necessary to conserve and use genetic resources (Giraldo et al., 2010).

It is vital to characterize for domestication and collection of the elite and promising genotypes of the country bean with high yielding potentialites for food and fodder purposes. Thus, the objective of the current study was to analyze the lablab germplasms based on their qualitative and quantitative morphological features related yield using multivariate analysis tools and identifiy divergent, and superior germplasms.

## 2 Materials and methods

### 2.1 Plant materials

The plant materials evaluated in this study consisted of 50 *Lablab purpureus* germplasm along with their passport data are shown in Table 1. Germplasm consisted of 30 accessions collected from the Plant Genetic Resource Centre (PGRC) of Bangladesh Agriculture Research Institute (BARI) and ten released bean varieties gathered from the same institute. The remaining ten germplasm were collected from farmers from different districts of Bangladesh. All the germplasm were during 2017 and used in experimentation from 2017 to 2020..

**Table 1:** List of 50 *Lablab purpureus* germplasm and their passport data.

Serial No	Name of the Germplasm	Collection District	Donor name	Collection Year	Longitude and latitude
Gene Bank accessions of *Lablab purpureus* conserved at PGRC, BARI					
**1**	BD-10798	Gopalganj	Akhter Mirdha	31-03-2014	Not known
**2**	BD-10799	Gopalganj	Enayet Shaikh	31-03-2014	Not known
**3**	BD-10800	Gopalganj	Enayet Shaikh	31-03-2014	Not known
**4**	BD-10801	Bhola	Jashim Sajwal	20-02-2014	Not known
**5**	BD-10802	Bhola	Arif Mahmood	20-02-2014	Not known
**6**	BD-10803	Bhola	Arif Mahmood	20-02-2014	Not known
**7**	BD-10804	Bhola	Sanker	20-02-2014	Not known
**8**	BD-10805	Bhola	Nitai Bapari	20-02-2014	Not known
**9**	BD-10806	Bhola	Liton Tofalli	20-02-2014	Not known
**10**	BD-10807	Bhola	Iqbal Hossain	20-02-2014	22°05.580' N 90°47.308'E
**11**	BD-10808	Bhola	Kamal Hossain	20-02-2014	22°06.025'N 90°45.745'E
**12**	BD-10809	Bhola	Kamal Hossain	20-02-2014	22°06.025'N 90°45.745'E
**13**	BD-10811	Bhola	Abul Kashem Farazi	21-02-2014	Not known
**14**	BD-10812	Bhola	Abul Kashem Farazi	21-02-2014	Not known
**15**	BD-10813	Bhola	Abul Bashar	21-02-2014	Not known
**16**	BD-10814	Bhola	Mr. Farid Maji	21-02-2014	Not known
**17**	BD-10815	Bhola	Md. Alamgir Hossain	21-02-2014	Not known
**18**	BD-10816	Bhola	Md. Alamgir Hossain	21-02-2014	Not known
**19**	BD-10818	Nilphamari	Md. Alam	29-05-2014	26°06.166' N 88°48.483'E
**20**	BD-11087	Bogura	Md. Shamsul	19-03-2015	Not known
**21**	BD-11088	Jhalakathi	Alpana Mistri	10-12-2014	22°44.170' N 90°11.124'E
**22**	BD-11089	Jhalakathi	Alpana Mistri	10-12-2014	22°44.170' N 90°11.124'E
**23**	BD-11090	Pirojpur	Md. Monir Hossain	09-12-2014	22°30.440' N 89°57.50' E
**24**	BD-11091	Pirojpur	Md. Monir Hossain	09-12-2014	22°30.440' N 89°57.502'E
**25**	BD-11092	Pirojpur	Md. Shahidul Islam Kazi	09-12-2014	22°34.350' N 89°31.650'E
**26**	BD-11093	Chhattogram	Jamal Hossain	30-08-2013	Not known
**27**	BD-11095	Chhattogram	Jamal Hossain	30-08-2013	Not known
**28**	BD-11097	Satkhira	Md. Khalirul Alam	14-09-2013	Not known
**29**	BD-11098	Jessore	Md. Harun Mia	13-09-2013	Not known
**30**	BD-11099	Munshiganj	Momtaz Begum	04-12-2014	Not known
Commercially released and locally collected *Lablab purpureus* varieties					
**31**	BARI Sheem-1	Released in 1996 by Bangladesh Agricultural Research Institute (BARI), Gazipur			
**32**	BARI Sheem-2	Released in 1996 by Bangladesh Agricultural Research Institute (BARI), Gazipur			
**33**	BARI Sheem-3	Released in 2006 by Bangladesh Agricultural Research Institute (BARI), Gazipur			
**34**	BARI Sheem-4	Released in 2007 by Bangladesh Agricultural Research Institute (BARI), Gazipur			
**35**	BARI Sheem-5	Released in 2009 by Bangladesh Agricultural Research Institute (BARI), Gazipur			
**36**	BARI Sheem-6	Released in 2011 by Bangladesh Agricultural Research Institute (BARI), Gazipur			
**37**	BARI Sheem-7	Released in 2011 by Bangladesh Agricultural Research Institute (BARI), Gazipur			
**38**	BARI Sheem-8	Released in 2015 by Bangladesh Agricultural Research Institute (BARI), Gazipur			
**39**	IPSA Sheem-2	Released in 1991 by Bangabandhu Sheikh Mujibur Rahman Agricultural University (BSMRAU)			
**40**	Khisamoti	Locally grown *Lablab* variety			
**41**	Rifa	Locally grown *Lablab* variety			
**42**	Goal Goda	Locally grown *Lablab* variety collected from Sylhet			
**43**	Nodi	Locally grown *Lablab* variety			
**44**	Noldoc	Locally grown *Lablab* variety			
**45**	Ali	Locally grown *Lablab* variety collected from Jessore			
**46**	Laluri	Locally grown *Lablab* variety collected from Moulovibazar			
**47**	Mostofa	Locally grown *Lablab* variety collected from Cumilla			
**48**	Kaloputi	Locally grown *Lablab* variety collected from Hatazari, Chhattogram			
**49**	Chanchal	Locally grown *Lablab* variety collected from Jessore			
**50**	BARI Sheem-9	Released in 2017 By Bangladesh Agricultural Research Institute (BARI), Gazipur			

### 2.2 Experimental site and field experimentation

The field experiment was held at the Field Laboratory of the Department of Entomology, Bangladesh Agricultural University, Mymensingh 2202, Bangladesh, located 24.75” N latitude 90.5” E longitude at a mean elevation of 7.9–9.1m above sea level. The study was laid out in a Randomized Complete Block Design (RCBD) with three replications. The dimension of the field was 20 × 8m. The area was divided into 50 plots of 1.5 m × 1.5 m each. The plot-to-plot distance was kept at 30cm to properly facilitate intercultural and data collection operations. Seeds were soaked overnight, and ten seeds were placed in each pit. After germination, excess seedlings were uprooted from each pit, keeping three to four healthy seedlings per pit. Finally, when the seedlings were established, we keep two plants in each pit by removing excess plants to ensure keeping uniform plants (2/pit, i.e., approx. 30,000 plants/ha) throughout the experimental plot. All the horticultural practices, including fertilization, were done as per suggested the bean cultivation technology (Azad et al., 2017). The experiments were repeated for two consecutive years from 2018-2019 and 2019 to 2020.

### 2.3 Morphological traits

The morphological characterization of the *L. purpureus* germplasm was done based on qualitative and quantitative traits of the leaf, flower, pod, and seed of the *Lablab* bean. The data on different qualitative and quantitative traits were collected as per the previous reports of descriptors of dolichos bean (Byregowda et al., 2015). Sixteen different qualitative characteristics, including cotyledon color (CC), leaf color (LC), leaf shape (LS), vein color (VC), seed shape (SS), seed color (SeC), stem color (StC), growth habit (GH), pod color (PC), pod beak (PB), pod constriction (PCo), pod curvature (PCu), standard petal color (SPC), wing petal color (WPC), keel petal color (KPC) and flower color (FC) were recorded (Table S1 and S2).

On the other hand, seventeen quantitative traits, including 10-seed weight (SW), 10-seed volume (SV), seed length (SL), seed width (SeW), single leaflet area (SLA), leaflet width (LW), leaflet length (LL), leaf length (LeL), average pod weight (APW), pod length (PL), pod width (PW)), plant height (PH), pod thinness (PT), no. of flower per spike (NFPS), spike length (SpL), days to first flowering (DFF), and total no. of spike per plant (TNSPP) were evaluated as per the descriptors of dolichos bean mentioned earlier (Byregowda et al., 2015) (Table S4). The leaves and leaflets were sampled during the vegetative development stage of the lablab plant. The seed volume was measured by the water displacing method placed in a measuring cylinder during the overnight soaking before germination. The plant height was measured at the final harvest by measuring the length of the highest vein. For each trait, three leaves/pods/flowers were samples from three plants, and the average was measured. The data gathered were organized in a matrix for subsequent use with Microsoft Excel 2019 software (https://www.microsoft.com).

### 2.4 Statistical analysis

Both qualitative and quantitative morphological characters were considered to assess the diversity. The XLSTAT software was used for multivariate analysis (Addinsoft, www.xlstat.com). Principal Component Analysis (PCA) was performed to identify groups and find the axes and attributes that contributed significantly to the variance using the similarity matrix. Two-dimensional scatter plots were created using the first two principal components, which accounted for the most variance. Ward's minimal variance approach as a clustering algorithm (Williams, 1976) and squared Euclidean distances as a measure of dissimilarity were used in Agglomerative Hierarchical Clustering (AHC) (Ward, 1963).

Different descriptive statistics such as range, mean, and standard deviation of various quantitative characters and frequency percentage of qualitative characters were also calculated. One-sample variance test was performed to check the null hypothesis and whether the variation is significant or not, and Pearson's correlation matrix was also measured to determine the correlation between variables.

## 3 Results

### 3.1 Qualitative traits

**Figure 1:**
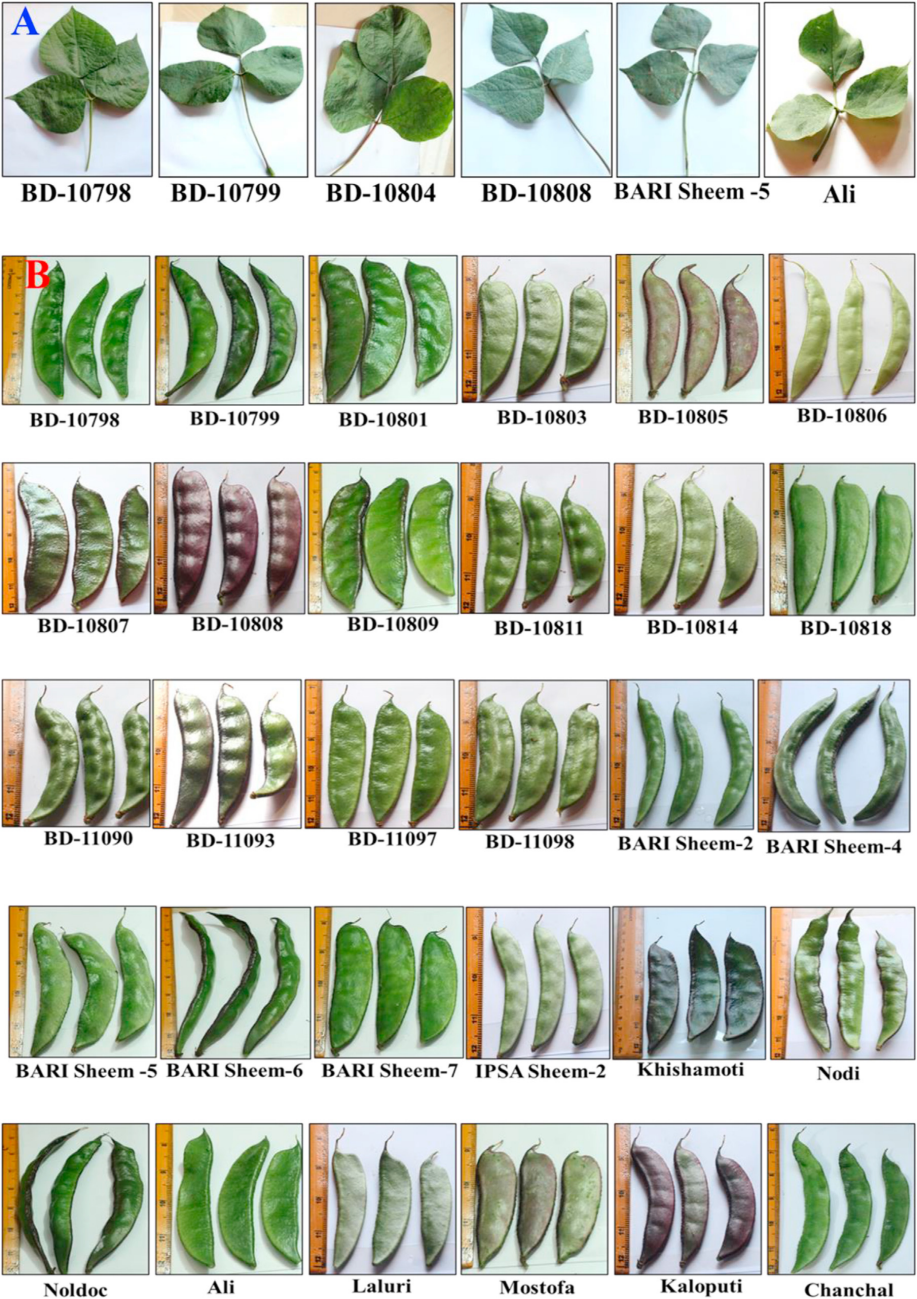
Morphological diversity for size, shape, color, length of leaves (A) and pods (B) among the 50 evaluated *Lablab* germplasm from Bangladesh. The name below each type represents the name of the respective germplasm identity.

**Figure 2:**
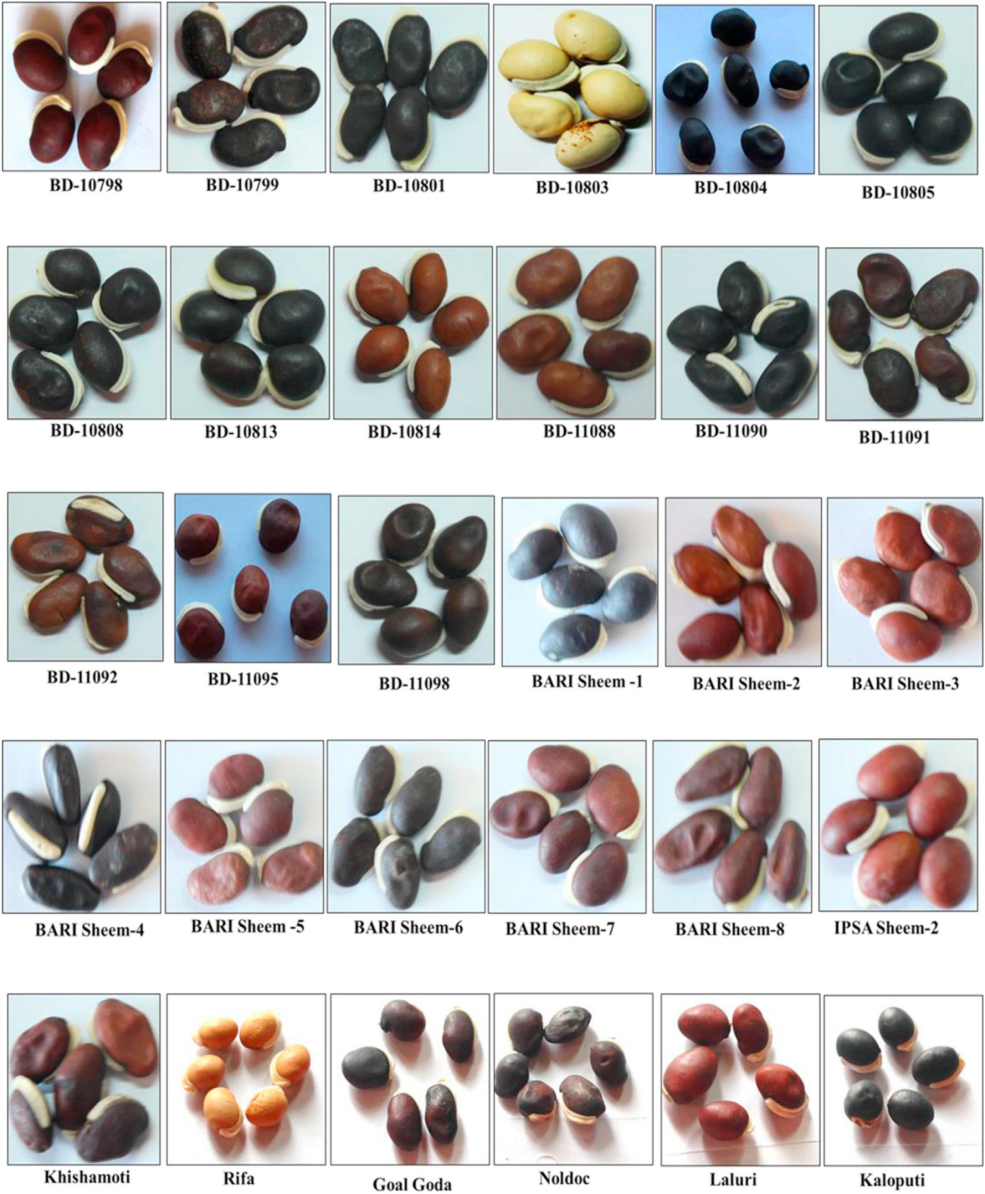
Morphological diversity for size, shape, and color of seed characteristics among the 50 evaluated *Lablab* germplasm collected from Bangladesh. The name below each type represents the name of the respective germplasm identity.

**Figure 3:**
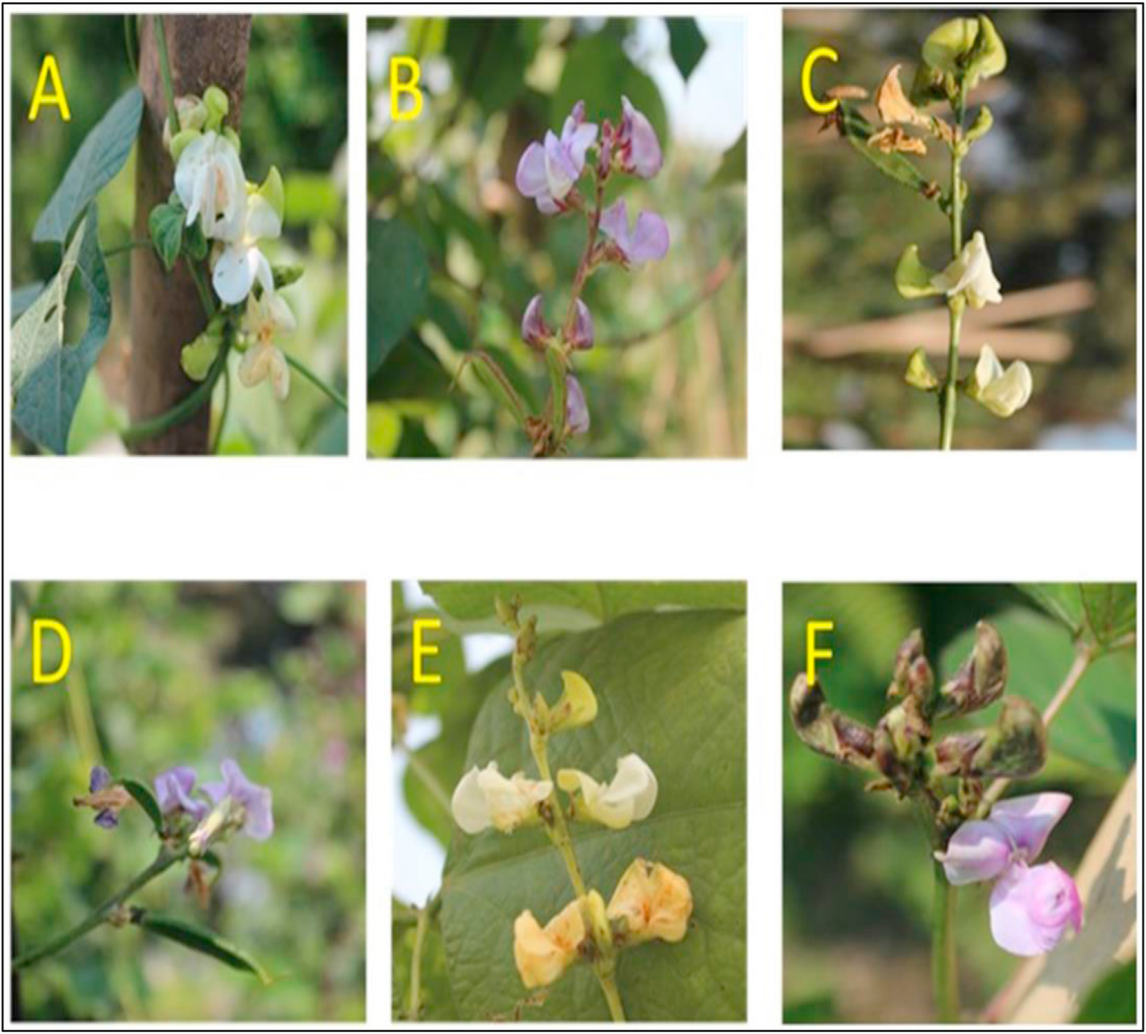
Representative flower color (FC) diversity among the 50 evaluated *Lablab* germplasm from Bangladesh (A) Flower of BD-10798 (white); (B) Flower of BD-10804 (Light purple); (C) Flower of BD-10814 (White); (D) Flower of Ali (Pink); (E) Flower of BD-11088 (White); (F) Flower of BD-11092 (Light purple).

The qualitative traits of the evaluated *Lablab* germplasm are shown in Table S1, and the conversion value of the individual traits is shown in Table S2. The leaf, pod, seed, and flower characteristics diversity is shown in Figures 1, 2, and 3. The relative percent frequency for the qualitative traits is shown in Figure S1. Our results exhibited significant variation among the features studied in the *Lablab* germplasms.

The cotyledon color was green in 21 germplasm (42% of the total), and the remaining 29 germplasm had light green cotyledon color (58%). It was found that the color of the leaves of the collected germplasm was mainly green (dark green, green, and light green leaves were 42%, 32%, and 24%, respectively). Most germplasms (49 of 50) were with ovate leaf shape and indeterminate type growth habit (98%). Vein color was also mostly green (dark green, green, and light green veins were 22%, 14%, and 30%, respectively), while the remaining germplasm had light purple (20%) or purple (14%) vein color. Stem colors also had similar types. Seven types of seed shape were identified in the evaluated germplasm where flat oval (32%), oval (26%), and round (26%) seed types constituted the significant variations. The seed colors of the collected germplasm are mainly black (56%) and brown (32%). Great diversity in pod color was identified (thirteen types were recorded). Green, dark-green, and light-green constitute the major types with 24%, 18%, and 32% of the total variation, while the remaining germplasm had purple or purple border-colored pods (26% of the total). As for pod beak and constriction, 36 germplasm had a short beak and straight pods (72%), the remaining germplasm had a long beak (26%), slight to moderate constricted (26%) pods. The majority of germplasm had little to moderately curved pods (94%), and a few had straight pods (6%). In the case of the standard, wing, and petal color of flowers, six types of color variation were recorded where white (30–36%), purple (26–34%), and light purple (22–28%) are the major types. Regarding flower color, six kinds, namely white (34%), light purple (18%), light violet (14%), pink (20%), purple (12%), and very light violet (2%) were recorded among the collected *Lablab* germplasm (Figure S1).

### 3.2 Quantitative traits

The quantitative traits of the evaluated *Lablab* germplasm are shown in Table S4. We have found that the collected germplasm varied considerably according to their characters. Significant genetic variations (p = 0.05, one-sample variance z-test) in descriptive statistics of the fifteen quantitative morphological traits except for seed weight and volume were found (Table 2).

**Table 2:**
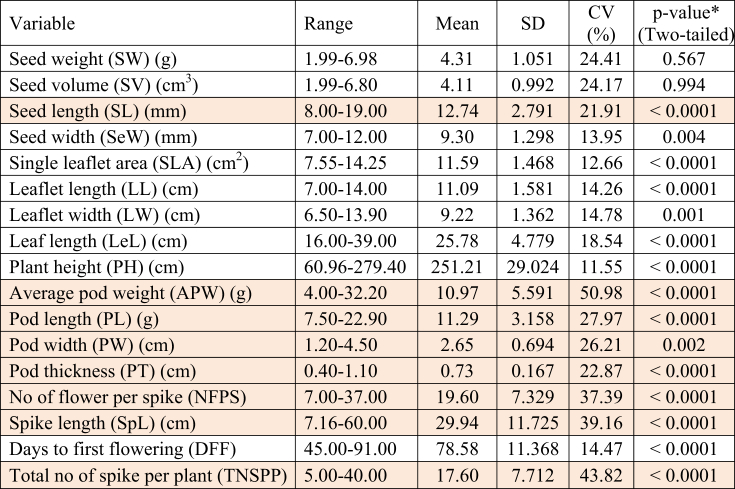
The descriptive statistics and analysis of seventeen quantitative morphological traits of *Lablab* germplasm from Bangladesh.

Traits having more than >20.00% higher CV value are marked with orange color.

∗**=** significance at alpha 0.05 of one-sample variance z-test.

### 3.3 Seed-related traits

While considering 10-seeds weight, the mean was found to be 4.31 g with the range of 6.98 to 1.99 g, SD was 1.051 g, and CV was 24.41% (Table 2). The lowest seed weight was found in Rifa, and the highest was observed in BD-10808 (Table S4). The average 10-seeds volume (cm^3)^ was found 4.11 cm^3^ with a range of 6.80 to 1.99 cm^3^, SD was 0.992 cm^3,^ and CV was 24.17%. The lowest seed volume was recorded in the Rifa cultivar, and the highest was observed in BARI Sheem-9. However, the seed weight and volume variation were not significant, but seed length and width varied significantly (as per the *z*-test, Table 2). The average seed length and width were 12.74mm (range 19.00–8.00), CV was 21.91% and 9.30mm (range 12.00–7.00), and CV was 13.95%, respectively (Table 2). The lowest seed length and width were recorded in BD-11095, BD-11097, and BD-10812, BD-11091, BD-11092, and BD-11098 germplasm, respectively, and the highest was observed in BD-10798, BD-11093, Ali, and Khisamoti, BARI Sheem-5, germplasm, respectively.

### 3.4 Leaf and plant-related traits

Significant variations were found among the *Lablab* germplasm about leaf and plant-related quantitative traits (Table 2). Leaflet length ranged from 14.0 to 7.0 cm with an average of 11.09 cm, SD was 1.581 cm, and CV was 14.26%. The highest leaflet length records in BD-10805, BD-10811, BD-10812, BD-11088, Nodi and *Mostafa germplasm*, and the lowest was BARI Sheem-1, respectively. The leaflet width ranged from 13.9 to 6.50 cm with an average of 9.22 cm, an SD of 1.362 cm and a CV of 14.78%. The highest leaflet width was recorded in BD-10804, and the lowest was in BARI Sheem-1.. Since leaflet length and width directly contribute to the leaflet area, the single leaflet area also showed similar trends. The highest single leaflet area width was recorded in BD-10804, and the lowest was in BARI Sheem-1 (Table S4).

The leaf length ranged from 39.0 to 16.0 cm with an average of 25.78 cm, SD was 7.779 cm, and CV was 18.54%. The highest leaf length was recorded in BD-10804, and the lowest was in BD-10798. The plant height of the most extended branch was also measured ranging from 279.40 (in Nodi) to 60.96 cm (in Rifa) with an average of 251.21 cm, SD was 29.02, and CV was 11.55 % (Table 2 and S4).

### 3.5 Pod-related traits

Pod-related characters varied significantly, and high variability in pod size was identified (Table 2). While considering the single pod, the mean weight was 10.90 g ranging from 32.20 to 4.00 g, with 50.98% CV. The lowest single pod weight was found in Rifa the highest in BD-10804. The pod length, width, and thickness also showed significant variation. The average pod length was 11.29 cm, ranging from 22.90 to 7.50 cm, SD was 3.158, and the average pod width was 2.65 cm, ranging from 4.5 to 1.2 cm. The highest pod length and width were recorded in Noldoc and BD-10815 germplasm, while the lowest was found in BD-10818 and BD-10806, respectively (Table S4). All these variations could be responsible for varying pod yield of the germplasm.

### 3.6 Flower-related traits

Flower-related quantitative traits in the *Lablab* germplasm also varied significantly (Table 2). On average, 19.60 flowers per spike were found with the range of 37.00 (highest in Mostafa) to 7.00 (lowest in Chanchal), SD was 7.329, and CV was 37.39%. The spike length was also measured, with a mean of 29.94 cm and a range of 7.16–60.00 cm. The lowest spike length was found in Noldoc, and the highest was observed in BARI Sheem-3 of the collected germplasm (Table S4). In the case of the total number of spikes per plant character, a great deal of diversity was observed. On average, 17.6 total spikes per plant were found, with a range of 5.00–40.00 having an SD of 7.712. The lowest total number of spikes per plant was found in the Chanchal cultivar, and the highest was observed in the BD-10805 accession among the collected germplasm. The days to the first flower in the plants, having a mean of 78.58 days ranging from 91.00 to 45.00 days, SD was 11.368 and CV was 14.47% (Table 2).

### 3.7 Analysis of correlation coefficients

Pearson's correlation coefficient analysis discovered significant relationships (p = 0.05), either positive or negative, among several variables in the *Lablab* germplasms (Tables 3 and 4).

**Table 3:** Correlation matrix for 16 qualitative morphological characters of *Lablab* germplasm from Bangladesh∗.

Variables	CC	LC	LS	VC	SS	SeC	StC	GH	PC	PB	Pco	Pcu	SPC	WPC	KPC	FC
CC	1.00															
LC	**-0.44**	1.00														
LS	-0.17	-0.08	1.00													
VC	**-0.51**	**0.57**	-0.24	1.00												
SS	0.21	-0.24	0.00	-0.22	1.00											
SeC	-0.23	**0.36**	0.10	**0.34**	-0.22	1.00										
StC	-0.17	-0.01	-0.24	0.10	-0.08	-0.01	1.00									
GH	-0.12	-0.14	0.02	-0.06	0.08	-0.10	-0.15	1.00								
PC	0.07	0.02	-0.13	0.00	0.23	-0.14	-0.17	0.10	1.00							
PB	0.15	-0.06	-0.09	-0.24	-0.03	-0.10	0.19	-0.24	-0.06	1.00						
Pco	0.24	-0.21	0.18	-0.27	-0.13	-0.02	-0.13	0.08	0.07	-0.07	1.00					
Pcu	0.09	-0.02	**-0.32**	0.00	-0.06	-0.23	0.09	0.05	0.07	0.03	0.12	1.00				
SPC	**0.42**	-0.11	-0.18	-0.12	0.21	-0.19	**-0.38**	0.18	**0.42**	-0.16	0.16	0.27	1.00			
WPC	**0.42**	-0.11	-0.18	-0.12	0.21	-0.19	**-0.38**	0.18	**0.42**	-0.16	0.16	0.27	**1.00**	1.00		
KPC	**0.37**	-0.16	-0.15	-0.17	**0.31**	-0.17	**-0.37**	0.15	**0.39**	-0.10	0.22	0.14	**0.91**	**0.91**	1.00	
FC	**0.34**	**-0.32**	-0.09	-0.22	**0.40**	-0.27	-0.18	0.09	0.07	-0.05	0.16	0.11	**0.36**	**0.36**	**0.34**	1.00

Values in bold are significant at Probability 0.05 (p ≤ 0.05).

**∗**The 16 qualitative morphological traits are cotyledon color (CC), leaf color (LC), leaf shape (LS), vein color (VC), seed shape (SS), seed color (SeC), stem color (StC), growth habit (GH), pod color (PC), pod beak (PB), pod constriction (PCo), pod curvature (PCu), standard petal color (SPC), wing petal color (WPC), keel petal color (KPC) and flower color (FC).

**Table 4:** Correlation matrix for 17 quantitative morphological characters of *Lablab* germplasm from Bangladesh.

Variables	SW	SV	SL	SeW	SLA	LL	LW	LeL	PH	APW	PL	PW	PT	NFPS	SpL	DFF	TNSPP
SW	1.00																
SV	**0.84**	1.00															
SL	0.27	**0.29**	1.00														
SeW	**0.32**	0.26	**0.31**	1.00													
SLA	0.26	0.25	0.12	0.19	1.00												
LL	0.25	0.26	0.16	0.20	**0.96**	1.00											
LW	0.16	0.10	-0.03	0.07	**0.67**	**0.43**	1.00										
LeL	**0.29**	0.23	-0.17	0.18	**0.59**	**0.55**	**0.42**	1.00									
PH	0.19	0.16	-0.08	-0.08	0.10	0.09	0.06	-0.08	1.00								
APW	0.19	0.20	0.06	0.07	0.07	0.01	0.19	**0.36**	0.12	1.00							
PL	-0.13	-0.07	-0.01	0.07	0.14	0.14	0.09	**0.33**	0.08	**0.49**	1.00						
PW	-0.11	-0.14	-0.10	0.05	-0.03	-0.07	0.10	0.08	-0.01	**0.50**	0.15	1.00					
PT	**0.33**	0.25	0.10	0.02	0.04	0.13	-0.21	-0.13	0.03	-0.12	0.02	**-0.42**	1.00				
NFPS	0.23	**0.32**	-0.13	0.17	-0.04	-0.03	-0.06	-0.03	0.03	-0.07	**-0.28**	-0.03	-0.02	1.00			
SpL	0.19	0.19	-0.20	0.09	-0.05	-0.13	0.18	-0.01	-0.06	-0.01	**-0.37**	0.17	-0.25	**0.75**	1.00		
DFF	0.13	0.24	0.04	-0.21	0.03	-0.01	0.12	-0.16	0.09	-0.15	-0.11	**-0.32**	0.12	0.25	0.13	1.00	
TNSPP	0.23	0.22	-0.12	-0.04	0.15	0.14	0.12	0.04	0.04	**-0.36**	**-0.43**	-0.15	0.03	**0.52**	**0.40**	0.14	1.00

Values in bold are significant at Probability 0.05 (p ≤ 0.05).

**∗**The 17 quantitative morphological traits are 10-seed weight (SW), 10-seed volume (SV), seed length (SL), seed width (SeW), single leaflet area (SLA), leaflet length (LL), leaflet width (LW), leaf length (LeL), plant height (PH), average pod weight (APW), pod length (PL), Average pod width (APW), pod thickness (PT), no. of flower per spike (NFPS), spike length (SpL), days to first flowering (DFF), and total no. of spike per plant (TNSPP).

Among the qualitative traits, cotyledon color, the three petal-related traits (standard petal color, SPC, wing petal color, WPC, keel petal color, KPC), stem, pod, and flower color were found to be significantly associated with other traits (p ≤ 0.05) (Table 3).

All the three petal-related traits (SPC, WPC, KPC) were found showing very strong, positive but significant correlation (r = 1.00 and 0.91) with each other. However, they were found showing moderate and negative correlation with stem color (StC) and positive associated with pod color (PC) (Table 3, p ≤ 0.05). A strong, negative but significant association was found between cotyledon color (CC) and vein color (VC) (r =-0.51), the Leaf color (LC) is found positively correlated with vein color (VC) (r = 0.57). Others characters showed either positive or negative but weak to moderate assocaitetions as presented in Table 3.

A significant correlation ((p ≤ 0.05)), either positive or negative, was also found among some quantitative traits measured in the *Lablab* germplasm (Table 4). The seed weight (SW) is positively correlated to seed volume (SV) (r = 0.84). . Among the leaf-related quantitative traits, a strong positive and significant correlation was observed between the single leaflet area (SLA) and leaflet length (LL), leaflet width (LW), and leaf length (LeL) (r = 0.96, 0.67 and 0.59, respectively). Leaflet length (LL) positively correlated with leaflet width (LW) and leaf length (LeL) (r = 0.55). In case of pod related characteristics, the average pod weight (APW)) was found to have a positive and significant correlation with pod length (PL), pod width (PW), and leaf length (LeL) but a negative correlation with a total no. of spike per plant (TNSPP).

Finally, among various flower related quantitative traits, the spike length (SpL), and number of flower per spike (NFPS) showed strong and positive (r = 0.75) associations (Table 4, p ≤ 0.05). Others quantitative traits showed either positive or negative but weak to moderate assocaitetions as presented in Table 4. Overall, compared to qualitative traits showed strong associations than the qualitative traits (Table 4).

### 3.8 Principal component analysis (PCA)

Principal component analysis (PCA) was used using 16 qualitative and 17 quantitative morphological qualities to discover the distinct factors/components that strongly impacted the comprehensive indicators.

### 3.9 PCA analysis of the qualitative morphological traits

**Figure 4:**
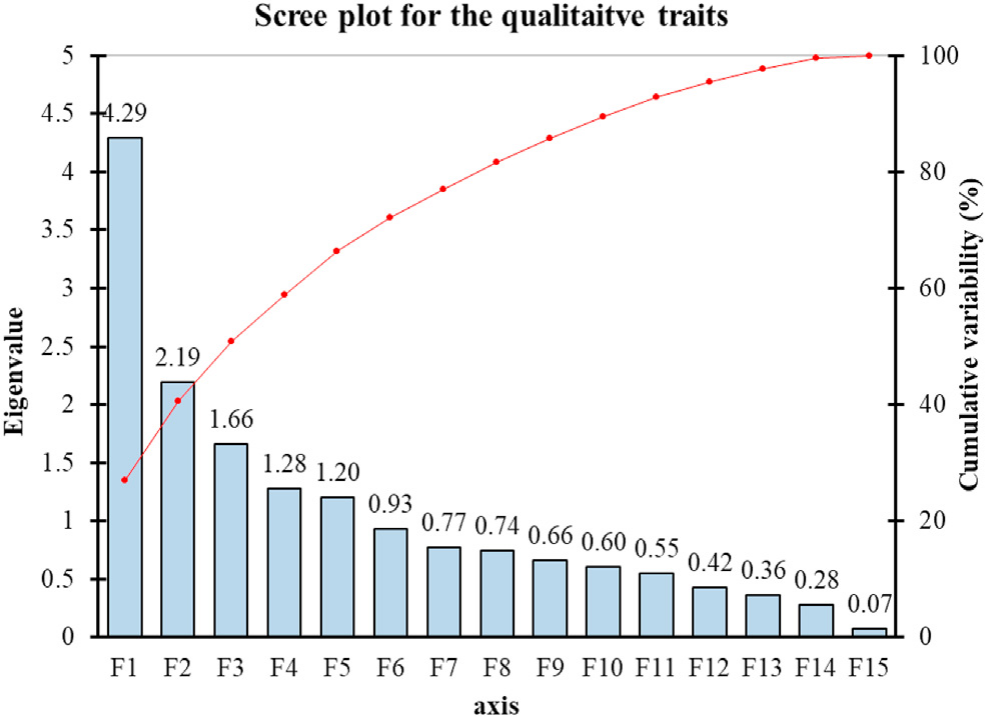
The scree plot shows the eigenvalues of different principal components (bars in the figure) and the percent cumulative variability (line in the figure) as shown by PCA of the *Lablab* germplasm based on qualitative morphological traits.

PCA identified that five principal components (F1, F2. F3. F4, and F5) significantly contributed 66.38% of the total variation in qualitative morphological traits. F1 was described as having the highest variance (26.83%) followed by F2, F3, F4, and F5 accounted for 13.68, 10.39%, 7.98, and 7.49% of the total qualitative morphological variation, respectively. The five components, therefore, were considered for further analysis. The Eigenvalues, contribution rate to variability, and cumulative contribution rate is shown in Table 5 and Figure 4.

**Table 5:**
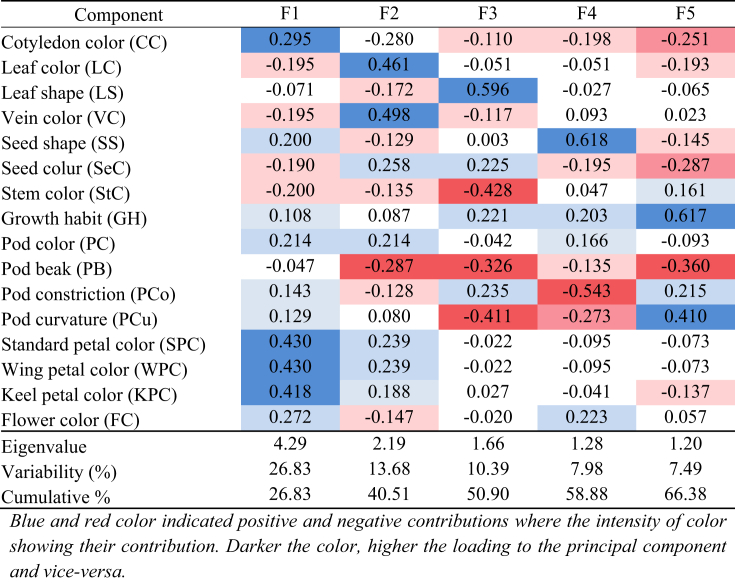
Eigenvalues, proportion of variability and cumulative rate of the 16 qualitative morphological characters of *Lablab* germplasm contributed to first six principal components (PCs).

**Figure 5:**
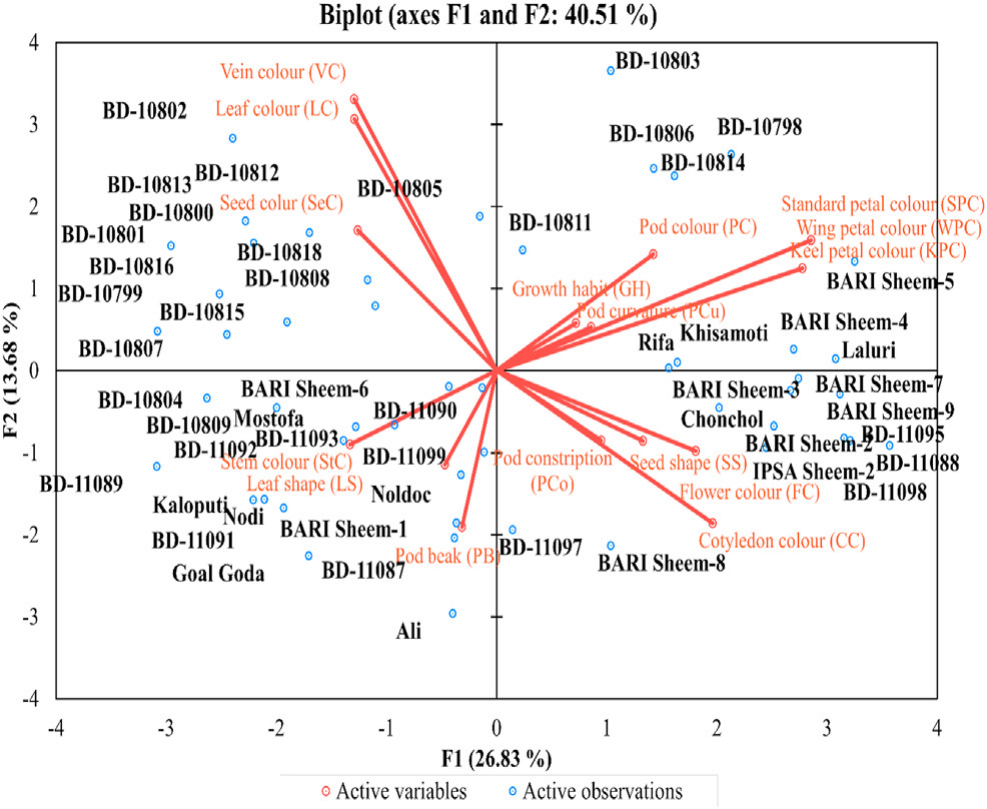
The biplot of 50 *Lablab* germplasm for principal components one (F1) and two (F2) of the qualitative morphological traits. The lines show the contribution (direction and magnitude) of the 16 quantitative morphological traits in the principal components F1 and F2.

The PCA and scree plot analysis (Figure 4) of qualitative traits showed that the Cotyledon color (CC), petal related traits (standard petal color, SPC, wing petal color, WPC, keel petal color, KPC) were strong. The pod color (PC), flower color (FC), pod constriction (PCo), and pod curvature (PCu) were moderately loaded in F1, suggesting that parameters included in F1 were mainly related to pod and flower characteristics. The F2 of PCA showed a strong positive contribution to Leaf color (LC), Vein color (VC), and moderate to all the 3-Pl color-related traits, pod color (PC), seed color (SeC), and flower color (FC) but negatively loaded to pod beak (PB) indicating that F2 as the main factor contributing plant (Leaf, vein, flower, seed) color. In F3, the eigenvalue was 1.66; leaf shape (LS) positively, but stem color (StC), pod beak (PB), and pod curvature (PCu) loaded negatively in F3 suggested that F3 is the crucial factor of plant structure. The F4 and F5 positively loaded to seed shape (SS) and growth habit (GH) with pod curvature (PCu), while showing negative loading to pod constriction (PCo) and pod beak (PB), indicating that these two components contributed to the seed and pod structure of the plant. Overall, the F1 and F2 components constitute 40.51% of the total qualitative morphological variations with plant, pod, and flower-related qualitative traits (Table 5, Figure 4). The direction and degree of contribution of the qualitative morphological characters in the different principal components are shown in Figure 5.

### 3.10 PCA analysis of quantitative morphological characters

**Figure 6:**
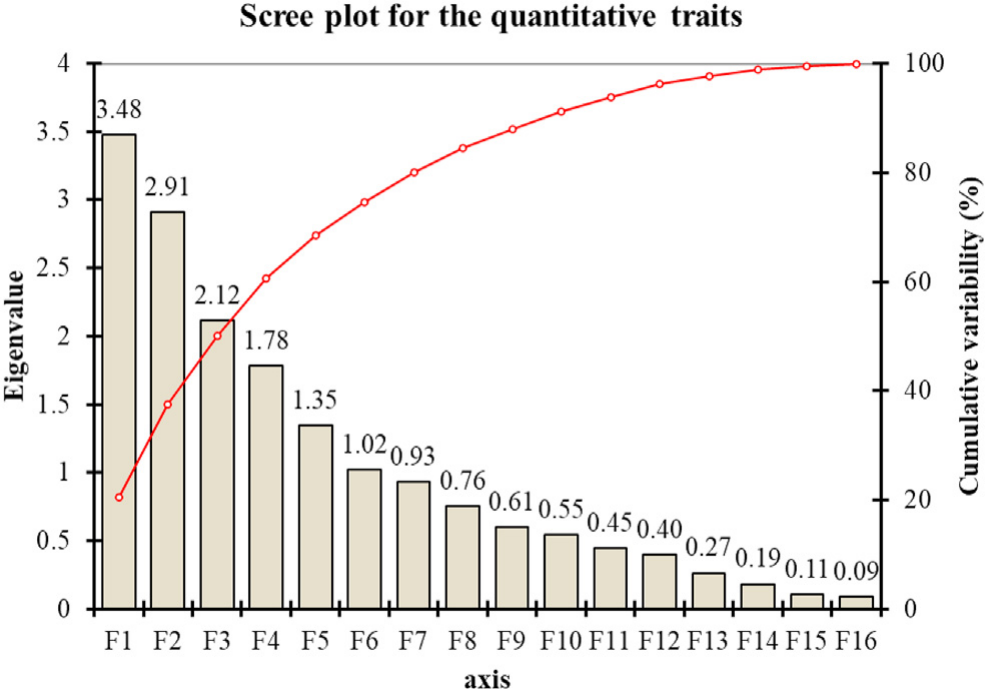
The scree plot shows the eigenvalues of different principal components (bars in the figure) and the percent cumulative variability (line in the figure) as shown by PCA of the *Lablab* germplasm based on quantitative morphological traits.

The Eigenvalues, contribution percent to variability, and the cumulative contribution rate of the first six principal components (F1, F2. F3. F4, F5, and F6) contributed to the quantitative morphological traits are shown in Figure 6. PCA analysis revealed that six of the fifteen principal components significantly contributed to nearly 75% of total quantitative variations. The six components were used for further analysis (Table 6, Figure 6). The first component (F1) possesses the highest variance (20.48%), followed by F2, which accounted for 17.12% while F3, F4, F5, and F6 accounted for 12.47%, 10.50%, 7.92%, and 6.02% of the total quantitative morphological variation, respectively (Table 6).

**Table 6:**
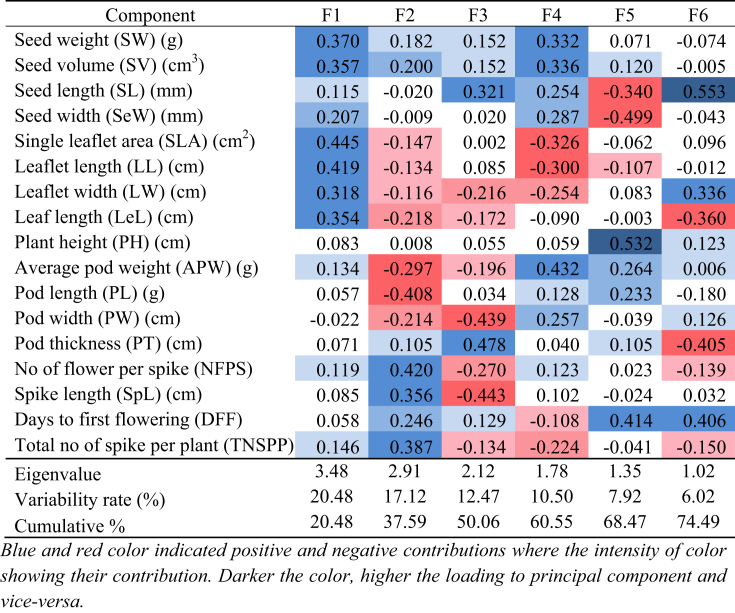
Eigenvalues, proportion of variability and cumulative rate of the 17 quantitative morphological characters of *Lablab* germplasm contributed to the first six principal components (PCs).

**Figure 7:**
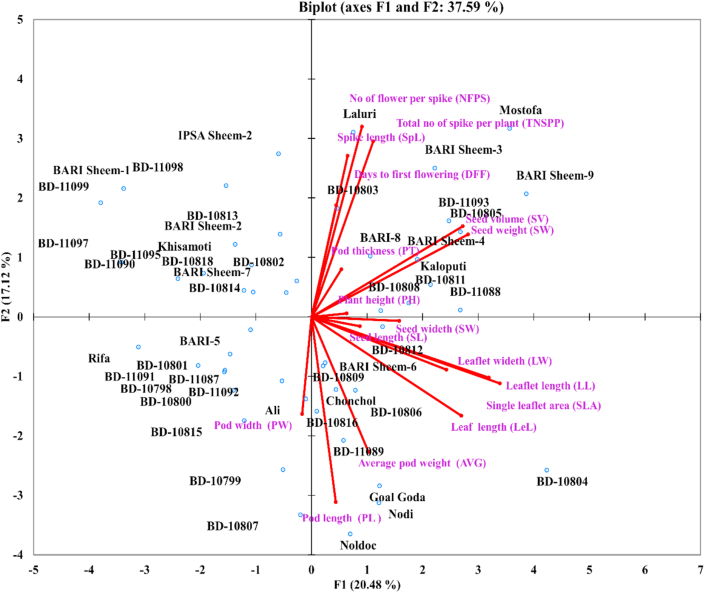
The biplot of 50 *Lablab* germplasm for principal components one (F1) and two (F2) for the quantitative traits. The lines show the contribution (magnitude and direction) of the 17 quantitative morphological traits in principal components F1 and F2.

PCA of quantitative traits exhibited that the seed weight, seed volume, single leaflet area, leaflet length, width, and leaf length were strongly loaded in the first principal component (F1), suggesting the F1 component is mainly related to leaf, seed, and flower characteristics. This component was positively contributed by all variables except pod width, whose contribution was ignorable (Table 6). The F2 of PCA is positively contributed by number of flower per spike, spike length, days to first flowering, the total number of spike per plant, seed weight, and volume components but negatively loaded to average pod weight, pod, and width and also to leaf and leaflet characters indicating that F2 as the main factor contributing flower, pod and leaf characteristics. F3 and F4 components contributed similarly to the seed and pod structure of the plant. F5 and F6 components showed strong positive load by plant height and seed length, suggesting the major factor contributing to plant and seed characteristics. Overall, the F1 and F2 components constitute 37.59% of the total quantitative morphological variations with seed, leaf, pod, and flower-related quantitative traits. The extent and direction of the contribution of the quantitative traits in the different principal components are shown in Figure 7.

### 3.11 Cluster analysis

**Figure 8:**
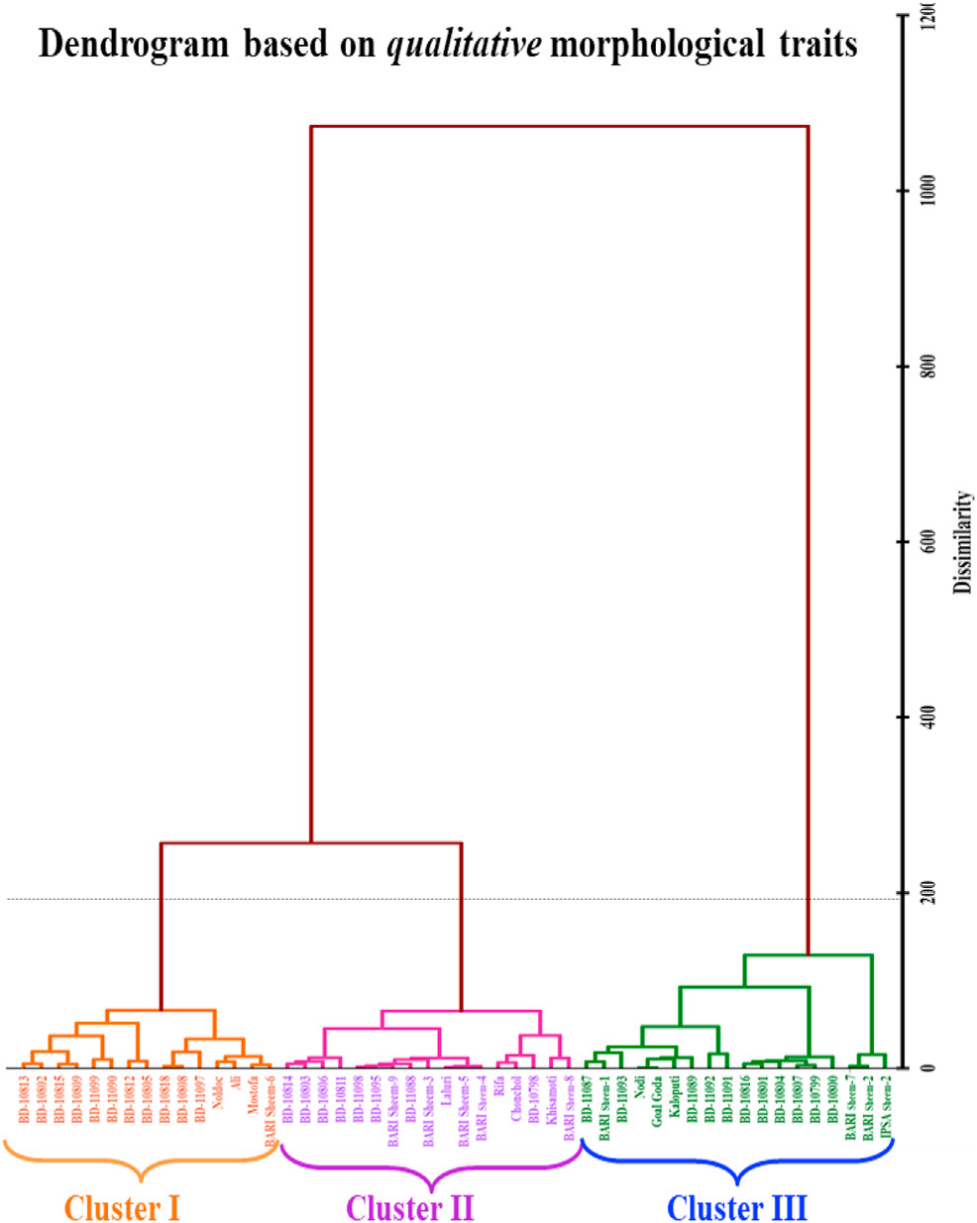
Agglomerative hierarchical clustering (AHC) dendrogram analysis using Euclidean distance into different clusters as per Ward method for *qualitative* morphological traits of 50 *Lablab* germplasms collected from Bangladesh.

**Figure 9:**
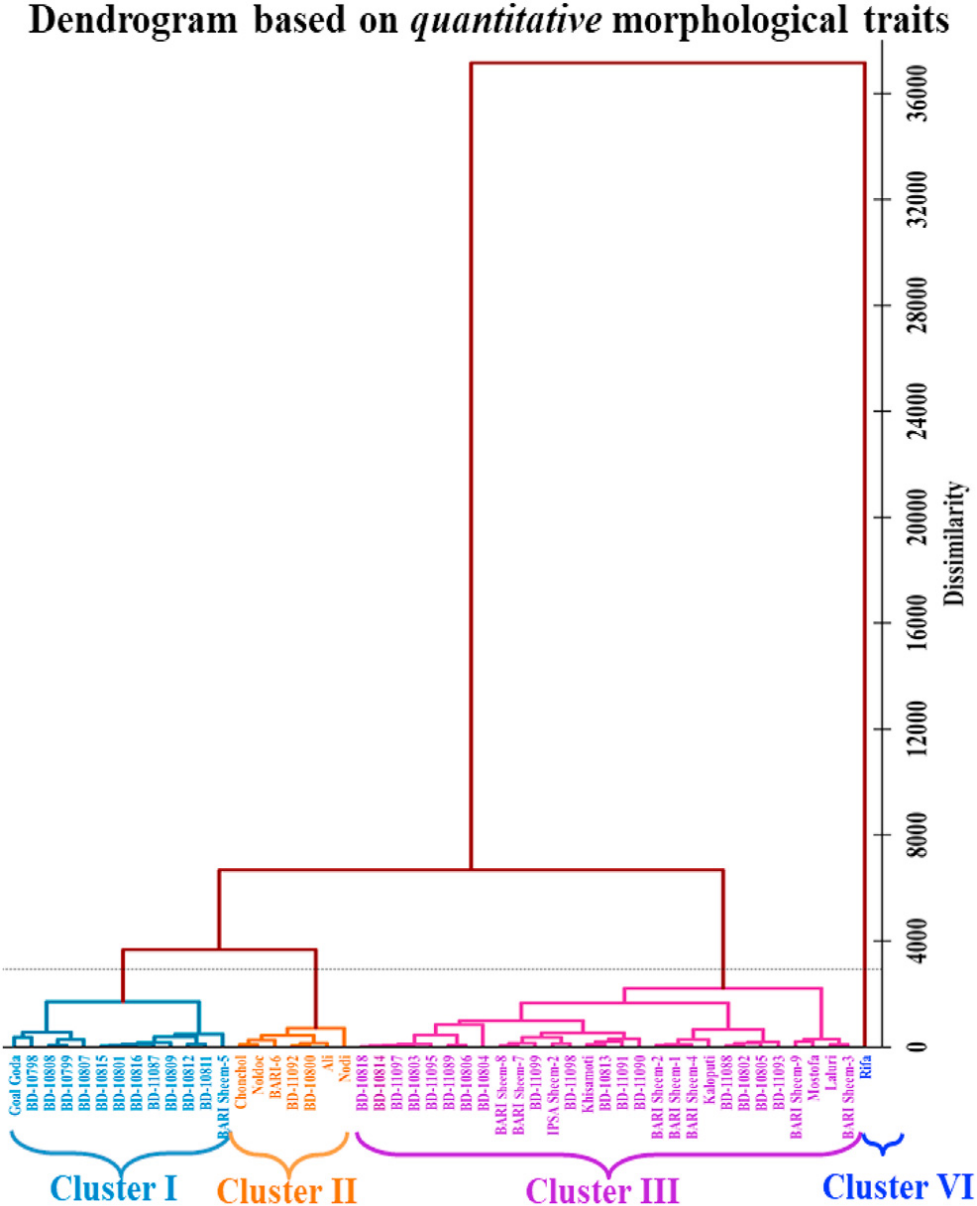
Agglomerative hierarchical clustering (AHC) dendrogram analysis using Euclidean distance into different clusters by the Ward method for *quantitative* morphological traits of 50 *Lablab* germplasm collected from Bangladesh.

The Ward technique was used to perform the agglomerative hierarchical clustering (AHC) analysis. The resulting dendrogram is displayed in Figures 8 and 9 for qualitative and quantitative morphological traits, for the evaluated 50 *Lablab* germplasm. The evaluated germplasms were grouped into 3 and 4 clusters based on qualitative and quantitative morphological traits, as listed in Table S3. Qualitative traits clustering results in four clusters, clusters I, II, and III including 15, 17, and 18 germplasm.

The quantitative traits clustered 13, 7, 29, and 1 germplasm to the cluster I, II, III, and VI, respectively. The Cluster III of quantitative traits grouping is the largest and includes 17 collected accessions; the other 12 were locally released varieties of country bean. Thus, grouping based on quantitative traits offers better clustering than qualitative ones. Based on clustering, we further analyzed the mean performance of different quantitative traits in each cluster and compared the mean of the total 50 germplasm presented in Table 7.

**Table 7:**
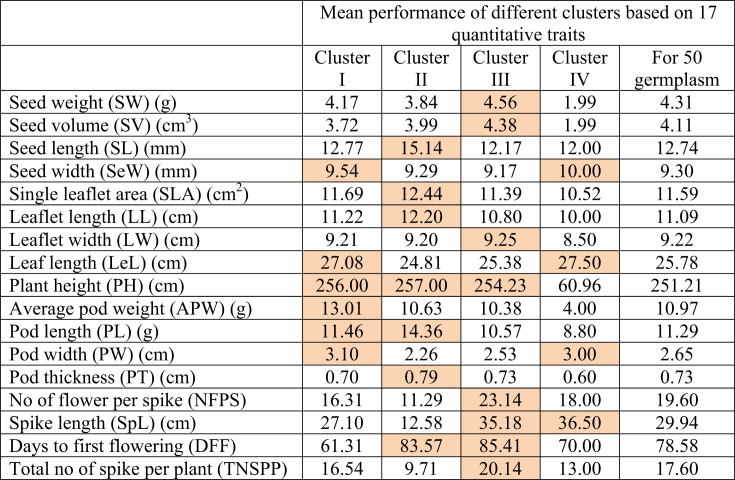
The mean performance of different 17 quantitative traits in each cluster along with mean of the evaluated 50 germplasm.

The orange-colored values indicate higher mean values in different clusters in comparison with the total mean of the 50 germplasm (sixth column).

Cluster I of quantitative traits grouping contained 13 germplasm of which 11 are accessions collected by PGRC, BARI, one released, and the other is a local variety. The main feature of this cluster is the higher mean values of seed width, leaf length, average pod weight, pod length, plant height, and pod width compared to other clusters and the total mean for 50 germplasm (Table 7). Cluster II of quantitative traits grouping contained seven germplasm, two of which are collected accessions, and the rest are either released or local varieties. This group has higher seed length, leaflet area, and length, plant height, pod length and thickness, and days to first flowering values. Cluster III containing 29 germplasms had higher mean values for the traits, mainly seed and flower-related. Cluster VI, having only one genotype Rifa, a cultivated local variety, possesses higher seed width, leaf length, pod width, and spike length than other clusters and a total mean of 50 germplasm evaluated (Table 7).

## 4 Discussion

### 4.1 Germplasm screening

Germplasm assessment and screening for desired features is a well-established crop breeding method to manage and utilize plant genetic resources effectively and efficiently (Nietsche et al., 2015). Diversity analysis is a critical step in determining genetic connections of the available genetic resources. For a long time, morphological features have been employed to examine relationships between plant genotypes and estimate their genetic diversity (Rai et al., 2010; Mohammadi and Prasanna, 2003). Morphological traits are comparatively easy to identify and differentiate, and do not require expert knowledge; published descriptor lists for most crop species are also publicly available. For many crop species, including *L. purpureus*, analyzing morphological features is a standard technique for measuring genetic variation. It is successfully employed on crops such as mungbean (*Vigna radiata*); black gram (*Vigna mungo*) (Ghafoor et al., 2001), sugarcane (*Saccharum officinarum*) (Sumbele et al., 2021); rice (*Oryza sativa*) (Qamar et al., 2012; Chakma et al., 2012; Dhakal et al., 2020) and also to wheat (*Triticum aestivum*) (Bechere et al., 1996).

The *L. purpureus* is a demandable legume crop, particularly in Asian and African countries. The Bangladesh-Indo region is a rich center of diversity for various crops. The *L. purpureus* germplasm collection and evaluation were reported from coastal areas (Shibli et al., 2021), Patuakhali (Ratna et al., 2021), and the south-western part (Hossain et al., 2014), Sylhet (Akter et al., 2017). A core collection of *L. purpureus* germplasms was developed at the Plant Genetic Resource Centre (PGRC), Bangladesh Agricultural Research Institute, Bangladesh, where 484 accessions were maintained (Islam et al., 2014). Although BARI released several *L. purpureus* variety, but their yield is low. Developing varieties and/or lines with high yielding capacity having stress tolerance and resistance to insects or diseases is always preferable to growers and breeders. In this connection, we collected 50 *L purpureus* local germplasm from Bangladesh and evaluated them based on their qualitative and quantitative morphological characteristics.

### 4.2 Morphological characterization and co-relation of morphological traits

We evaluated *L. purpureus* germplasm using qualitative and quantitative traits collected from Bangladesh. Analysis of qualitative traits revealed green color dominance in cotyledon, leaf, vein, and stem characteristics. Such dominance of green color over other color genotypes was also previously reported (Islam et al., 2014; Sultana, 2001). We have identified six flowers color types, whereas four types were reported earlier (Islam et al., 2014), indicating the presence of more variability among the germplasms. Pod color among the collected germplasms showed the highest variability, primarily green and their derivative types, purple or purple border-colored pods, slightly inconsistent with previous types (Islam et al., 2014; Islam, 2008) reddish and whitish pod color types were also reported. We did not record any such types in the present studies. However, it is consistent with studies by Sultana (2001) and Islam et al. (2010) where the dominance of green pod color was reported. Colorful flower and pigmentation in plants are vital attributes for pollinators to facilitate cross-pollination. Further, pigmentation chemicals are a diverse group of defense-related compounds that protect foliage from UV radiation, diseases, and insects (Freeman and Beattie, 2008). We found pods of various sizes, lengths, widths, thicknesses, and colors. The size and color of pods are vital characteristics in determining a crop's marketability. Cotyledon color showed a positive correlation with petal and flower color but negative relation with vein and leaf color. Leaf color showed positive relation to the vein and seed color. The petal color exhibited a positive correlation to pod color but negative relation to stem color. Thus, studies of qualitative morphological traits of plants justify germplasm characterization.

The pod yield of a crop is a complicated character and is the outcome of the action and interaction of numerous components. Hence, to develop the elite variety having higher yield or other characteristics, the association of pod yield or weight with other traits must be considered for the breeding program. The pod weight had a significant and positive correlation with the pods’ length and width and the leaves' length.. Similar findings have been previously reported for the number of grains per pod (Singh et al. (2011), the positive relationship between pod weight and length (Magalingam et al., 2013; Patil et al., 2017), between pod length and width (Upadhyay and Mehta, 2011), between pod length, and mean pod weight with pod width (Choudary et al., 2016). The pod length and width are found to have a significant negative correlation with number of flower per spike, spike length, total number of spike per plant, pod thickness, and days to first flowering (Table 6), which agrees with the previously reported by Asaduzzaman et al. (2014).

Seed yield results from numerous factors assessed at various stages of development (Savitha et al., 2008). Seed yield per plant was positively correlated with the parameters like number of pods/plant, number of pods/raceme, racemes/plant, 100 seed weight, the length of pod & seed, and number of seeds/pod (Kamotho, 2015). Similar observations were reported in pigeon pea (*Cajanus cajan*) (Bhadru and Acharya. 2010). Rai et al. (2011) found that various seed properties were significantly positively correlated with pod characteristics after analyzing 48 Indian *Lablab* genotypes. They also found a correlation between seed yield/plant and the number of pods, seeds, plant branches, and 100-seed weight. In the analysis of 20 *Lablab* from Bangladesh, Ali et al. (2005) also reported a strong positive connection between the seed yield per plant and the length and width of the pods. Other characteristics might be linked to traits like the number of seeds per plant, seed yield, the number of pods per plant, and 100-seed weight.

The presence of a high coefficient of variation (CV) (>20%) for seed length, average pod weight, pod length, pod width, pod thickness, number of flower per spike, spike length, and total number of spike per plant traits all of which are contributing to yield of the plant representing the greater degree of variability. Average pod weight (50.98%) had the highest coefficient of variation (CV), followed by the total number of spikes per plant characteristic (43.82%) (Table2), indicating that the material has the most variability that may be utilized for further improvement. Such a high degree of diversities was reported in inflorescence and pod characters of *L. purpureus* (Islam, 2008; Parmer et al., 2013; Islam et al., 2014; Hossain et al., 2014; Dutta et al., 2016; Shibli et al., 2021; Ratna et al., 2021), which are consistent with our present findings. The seed length showed high variability among the evaluated germplasm used in this study, which does not agree with previous studies by Savitha (2008) and Kamotho (2015) who reported low phenotypic variability in seed length due to genotypic variation. Because *Lablab* bean is native to Southeast Asia and has been brought to Africa and other tropical and subtropical countries. The great diversity observed in the traits in our examined germplasm might be related to a high degree of endemism. According to Savitha et al. (2008), the wide diversity in characteristics provides enough chance to choose genotypes based on the demand or type of breeding program. Several *Lablab* accessions of our studies may be selected for certain desired features. All these suggested presences of immense opportunities for yield enhancement by choosing genotypes with better pod-related characteristics for future endeavors.

### 4.3 Principal component analysis

The application of multivariate statistical analysis, such as PCA, can help to understand the relationship between variables. These might be useful for deducing the nature of attributes and reducing the complexity of data collection (Al-Sayed et al., 2012). PCA analysis of the seventeen quantitative traits revealed that the first six components contribute 74.49% of the total variation which is in agreement with previous reports of Islam et al. (2014) from Bangladesh who reported that the first six components of *Lablab* core collection contributed 73.74% of the total variation, also with the observations of Islam (2008) who reported that the first three principal components accounted for 75% of the total variation. Chowdhury and Mian (1996) reported a similar observation in field pea crop. The F1 and F2 biplot graphs of quantitative traits indicated that the pod weight had a strong positive correlation with pod length and width.

### 4.4 Cluster analysis

Cluster analysis is one of the popular statistical techniques to categorize objects in clusters having high similarities compared to other clusters. Because various sets of alleles may influence their characteristics and performance, the clusters will be helpful for future heterotic breeding (Twumasi et al., 2018). The analysis of the mean values in different clusters (Table 7) showed that none contained any germplasm with all the necessary traits that might be chosen and utilized in the breeding program. The different desirable traits are gathered in different clusters. We found that yield contributing traits (plant height, average pod weight, pod length, and width) are comparatively higher in clusters with 11 collected accessions and two varieties of *Lablab* germplasm (Cluster I). The largest group, Cluster III contains 29 germplasms and contributes to the traits like flower number, spike length, number of spikes per plant, and days to first flowering which indirectly contributes to the yield of bean plants. In Cluster II, seed, flower, and fruit-related traits are expressed differently compared to other clusters. The highest seed and pod length was found in Cluster II compared to other clusters and total mean performance but showed a more extended time required for the first flowering. Germplasms from Cluster I required the shortest days to first flowering suggesting germplasms from this group may be considered for developing short-durational varieties (Table 7). Thus, these germplasms could be used as parental sources for the country bean hybridization program for diversified uses.

These characteristics were reported to contribute primarily toward genetic divergence in the country bean germplasm that pod yield per plant had positive correlations with them (Shibli et al., 2021; Islam, 2008), which is consistent with our findings present study. Hybridization between different clusters with the highest genetic divergence as parental lines is suggested to develop a genotype or variety with preferable characters. As reported in rice crops, the presence of extensive variability and segregations of traits with high heterotic effects (Omar et al., 2012; Chakma et al., 2012; Dhakal et al., 2020), also in Country bean (Shibli et al., 2021; Islam, 2008). Thus, our results will benefit breeders in selecting potential parental lines to develop hybrid varieties by exploiting heterosis in the segregating generations.

## 5 Conclusion

The Country bean is an underutilized crop. This study has demonstrated considerable morphological diversity of qualitative and quantitative traits among the *Lablab* germplasm collected from Bangladesh. Both the qualitative and quantitative characteristics studied here showed inter-relationship among them. Based on eigenvalues from PCA, it is justifiable to say that color-related qualitative traits of cotyledon, leaf, vein, seed, flower, and petals contributed highly. The quantitative traits, e.g., the seed length, pod length, width, thickness, number of flowers per spike, spike length, and total number of spikes per plant, contributed highly to the total diversity of the *Lablab* germplasm. The average pod weight strongly correlated with pod length and width. The germplasm, BD-10804, BD-10807, Goal Goda, BD-11091, BD-10808, BD-10815, and BD-11089 produced higher pod weight with corresponding higher pod length, width, and thickness. Given that the characteristics listed above directly impact *Lablab* production, this heterogeneity across germplasm opens up the prospect of developing superior varieties with specialized features for food and feed. The significant phenotypic diversity in the germplasm studied is also encouraging for conservation. Because environmental factors strongly influence morphological features, molecular and other methods should be used for additional analysis.

## Declarations

### Author contribution statement

Rahima Khatun: Conceived and designed the experiments, Performed the experiments; Analyzed and interpreted the data; Wrote the paper.

Md. Imtiaz Uddin: Contributed reagents, materials, analysis tools or data;

Mohammad Mahir Uddin: Contributed reagents, materials, analysis tools or data;

Mohammad Tofazzal Hossain Howlader: Analyzed and interpreted the data; Contributed reagents, materials, analysis tools or data; Wrote the paper.

Muhammad Shahidul Haque: Conceived and designed the experiments, contributed reagents, materials, analysis tools or data; Wrote the paper.

### Funding statement

Muhammad Shahidul Haque was supported by 10.13039/501100020039Bangladesh Academy of Sciences [BAS-USDA PALS CR35].

### Data availability statement

Data included in article/supp. material/referenced in article.

### Declaration of interest’s statement

The authors declare no conflict of interest.

### Additional information

No additional information is available for this paper.

## Appendix ASupplementary data

5-Supplementary figures and Tables -Morphology paper -Ok 5-Supplementary figures and Tables -Morphology paper -Ok
