# Supplementary material for: Analysis of qualitative and quantitative morphological traits related to yield in country bean (Lablab purpureus L. sweet) genotypes
Source: Heliyon. 2022 Nov 22;8(12):e11631. doi: 10.1016/j.heliyon.2022.e11631 (PMC9718983; doi:10.1016/j.heliyon.2022.e11631)
Supplement: 5-Supplementary figures and Tables -Morphology paper -Ok [file mmc1.pdf]

Supplementary figures

Supplementary Figure S1. The frequency percent for the sixteen qualitative morphological traits observed in 50 Country bean germplasms.

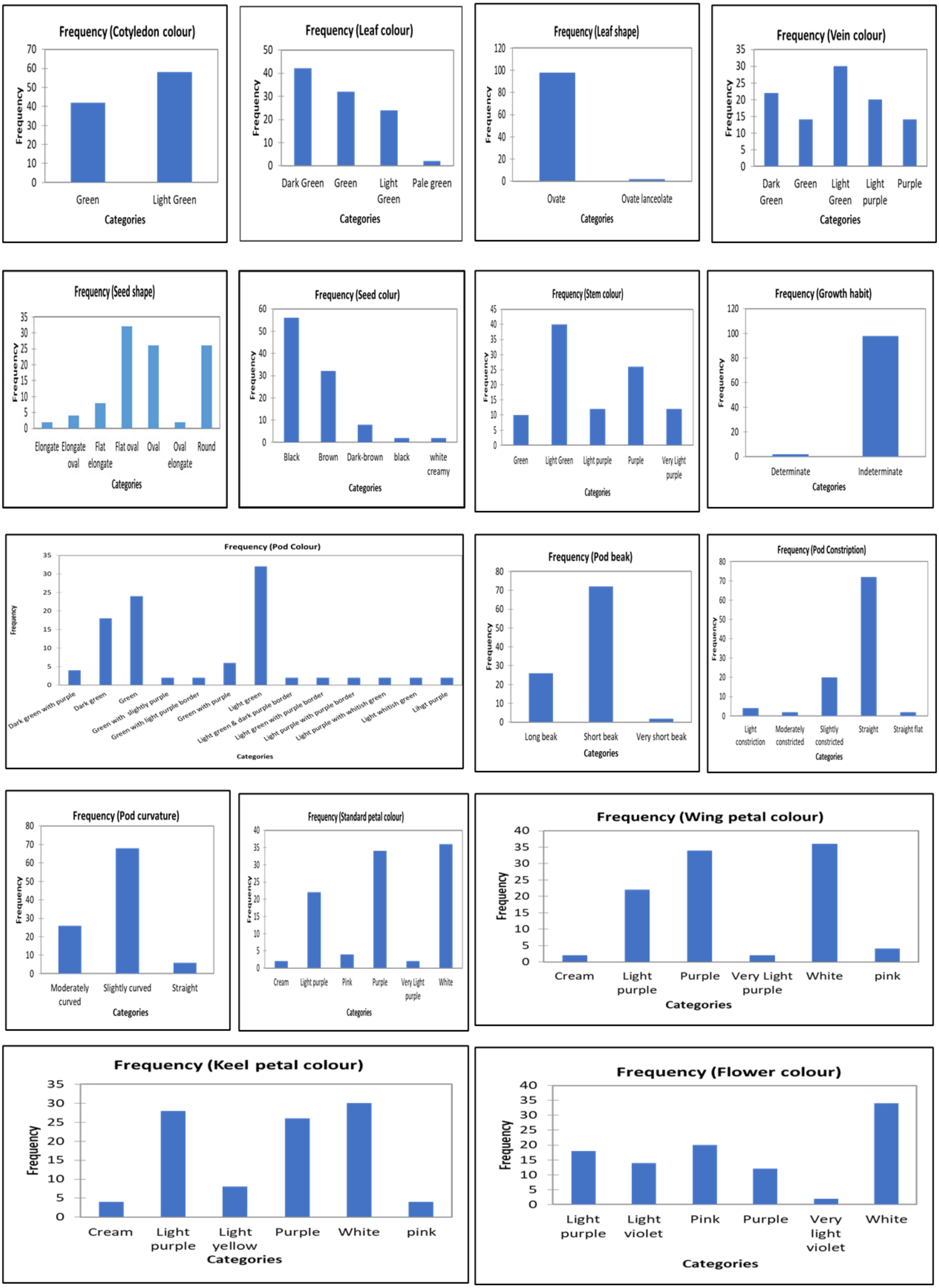
Frequency (Cotyledon colour)
Frequency
Green
Light Green
Categories
Frequency (Leaf colour)
Dark Green
Green
Light Green
Pale green
Frequency (Leaf shape)
Ovate
Ovate lanceolate
Frequency (Vein colour)
Dark Green
Light Green
Light purple
Purple
Frequency (Seed shape)
Elongate
Elongate oval
Flat elongate
Flat oval
Oval
Oval elongate
Round
Frequency (Seed colur)
Black
Brown
Dark-brown
black
white creamy
Frequency (Stem colour)
Light Green
Light purple
Very Light purple
Frequency (Growth habit)
Determinate
Indeterminate
Frequency (Pod Colour)
Dark green with purple
Dark green
Green with slightly purple
Green with light purple border
Green with purple
Light green
Light green & dark purple border
Light green with purple border
Light purple with purple border
Light purple with whitish green
Light whitish green
Lihgt purple
Frequency (Pod beak)
Long beak
Short beak
Very short beak
Frequency (Pod Constription)
Light constriction
Moderately constricted
Slightly constricted
Straight
Straight flat
Frequency (Pod curvature)
Moderately curved
Slightly curved
Straight
Frequency (Standard petal colour)
Cream
Light purple
Pink
Very Light purple
White
Frequency (Wing petal colour)
Very Light purple
pink
Frequency (Keel petal colour)
Light yellow
Frequency (Flower colour)
Light violet
Very light violet

Figure S1

Supplementary Tables

Table S1. Qualitative morphological traits of 50 *Lablab purpureus* germplasms

Table S2. The conversion value of the sixteen qualitative morphological traits of 50 *Lablab purpureus* germplasms

Table S3. Clustering of the 50 evaluated country bean germplasm based on qualitative and quantitative morphological traits as per agglomerative hierarchical clustering (AHC) analysis

Table S4. Mean of seventeen quantitative morphological traits of Lablab germplasm from Bangladesh

Table S1. Qualitative morphological traits of 50 *Lablab purpureus* germplasms

Serial no.	Name of the germplams	Cotyledon colour (CC)	Leaf colour (LC)	Leaf shape (LS)	Vein colour (VC)	Seed shape (SS)	Seed colur (SeC)	Stem colour (StC)	Growth habit (GH)	Pod colour (PC)	Pod beak (PB)	Pod constription (PCo)	Pod curvature (PCu)	Standard petal colour (SPC)	Wing petal colour (WPC)	Keel petal colour (KPC)	Flower colour (FC)
	**Gene Bank accessions of Lablab purpureus conserved at PGRC, BARI**																
1	**BD-10798**	Green	Pale green	Ovate lanceolate	Green	Oval	Brown	Green	Indeterminate	Green	Short beak	Slightly constricted	Straight	White	White	White	White
2	**BD-10799**	Light Green	Green	Ovate	Light purple	Flat oval	Black	Light purple	Indeterminate	Green with purple	Short beak	Slightly constricted	Slightly curved	Light purple	Light purple	Light purple	Purple
3	**BD-10800**	Light Green	Green	Ovate	Light purple	Flat oval	Black	Light Green	Indeterminate	Green with purple	Long beak	Slightly constricted	Moderately curved	Purple	Purple	Purple	Purple
4	**BD-10801**	Light Green	Green	Ovate	Green	Flat oval	Black	Purple	Indeterminate	Green with slightly purple	Short beak	Slightly constricted	Slightly curved	Purple	Purple	Purple	Light purple
5	**BD-10802**	Light Green	Green	Ovate	Green	Flat oval	Black	Light Green	Indeterminate	Green	Long beak	Moderately constricted	Moderately curved	Purple	Purple	Purple	Light purple
6	**BD-10803**	Light Green	Green	Ovate	Green	Oval	White creamy	Light Green	Indeterminate	Green	Long beak	Straight	Slightly curved	White	White	White	White
7	**BD-10804**	Green	Green	Ovate	Light purple	Oval	black	Purple	Indeterminate	Green with light purple border	Short beak	Slightly constricted	Slightly curved	Light purple	Light purple	Light purple	Light purple
8	**BD-10805**	Light Green	Green	Ovate	Light purple	Round	Black	Purple	Indeterminate	Light purple with purple border	Long beak	Straight	Slightly curved	Pink	pink	Pink	Purple
9	**BD-10806**	Light Green	Green	Ovate	Light purple	Oval	Black	Light Green	Indeterminate	Light whitish green	Long beak	Straight	Slightly curved	White	White	White	White
10	**BD-10807**	Light Green	Green	Ovate	Light purple	Flat oval	Black	Purple	Indeterminate	Green with purple	Short beak	Straight	Slightly curved	Purple	Purple	Light purple	Purple
11	**BD-10808**	Light Green	Green	Ovate	Purple	Oval	Black	Light purple	Indeterminate	Lihgt purple	Short beak	Straight	Slightly curved	Purple	Purple	Purple	Light violet
12	**BD-10809**	Light Green	Dark Green	Ovate	Light purple	Flat oval	Black	Light purple	Indeterminate	Green	Short beak	Straight	Slightly curved	Light purple	Light purple	Light purple	Purple
13	**BD-10811**	Light Green	Green	Ovate	Light purple	Oval	Black	Very Light purple	Indeterminate	Green	Short beak	Slightly constricted	Moderately curved	Pink	pink	pink	Light violet
14	**BD-10812**	Light Green	Green	Ovate	Purple	Oval	Black	Light purple	Indeterminate	Light purple with whitish green	Long beak	Straight	Slightly curved	Purple	Purple	Purple	Purple
15	**BD-10813**	Light Green	Green	Ovate	Green	Oval	Black	Very Light purple	Indeterminate	Green	Short beak	Slightly constricted	Moderately curved	Purple	Purple	Purple	Light purple

Table S1. (Continued)

Serial no.	Name of the germplams	Cotyledon colour (CC)	Leaf colour (LC)	Leaf shape (LS)	Vein colour (VC)	Seed shape (SS)	Seed colur (SeC)	Stem colour (StC)	Growth habit (GH)	Pod colour (PC)	Pod beak (PB)	Pod constription (PCo)	Pod curvature (PCu)	Standard petal colour (SPC)	Wing petal colour (WPC)	Keel petal colour (KPC)	Flower colour (FC)
16	**BD-10814**	Light Green	Green	Ovate	Green	Oval	Brown	Light Green	Indeterminate	Light green	Short beak	Straight	Slightly curved	White	White	Light yellow	White
17	**BD-10815**	Light Green	Dark Green	Ovate	Purple	Flat oval	Black	Very Light purple	Indeterminate	Dark green with purple	Short beak	Slightly constricted	Slightly curved	Purple	Purple	Light purple	Light purple
18	**BD-10816**	Light Green	Green	Ovate	Purple	Flat oval	Black	Light purple	Indeterminate	Dark green	Short beak	Straight	Slightly curved	Purple	Purple	Purple	Light purple
19	**BD-10818**	Light Green	Green	Ovate	Green	Round	Black	Very Light purple	Indeterminate	Light green	Short beak	Straight	Slightly curved	Purple	Purple	Light purple	Light violet
20	**BD-11087**	Green	Dark Green	Ovate	Light Green	Flat oval	Black	Light Green	Indeterminate	Dark green with purple	Short beak	Straight flat	Moderately curved	Purple	Purple	Purple	Light violet
21	**BD-11088**	Green	Dark Green	Ovate	Light Green	Flat elongate	Brown	Light Green	Indeterminate	Light green	Short beak	Straight	Slightly curved	White	White	Light yellow	White
22	**BD-11089**	Light Green	Dark Green	Ovate	Light purple	Flat oval	Black	Purple	Indeterminate	Dark green with purple	Short beak	Straight	Moderately curved	Light purple	Light purple	Light purple	Light purple
23	**BD-11090**	Light Green	Light Green	Ovate	Light Green	Round	Black	Very Light purple	Indeterminate	Green	Short beak	Straight	Moderately curved	Purple	Purple	Light yellow	Light purple
24	**BD-11091**	Light Green	Dark Green	Ovate	Purple	Flat elongate	Black	Purple	Indeterminate	Dark green with purple	Short beak	Straight	Moderately curved	Very Light purple	Very Light purple	Cream	Light violet
25	**BD-11092**	Light Green	Light Green	Ovate	Light purple	Flat elongate	Brown	Very Light purple	Indeterminate	Dark green	Short beak	Light constriction	Slightly curved	Light purple	Light purple	Light purple	Very light violet
26	**BD-11093**	Green	Dark Green	Ovate	Purple	Flat oval	Black	Purple	Indeterminate	Light green with purple border	Long beak	Straight	Moderately curved	Purple	Purple	Purple	Light violet
27	**BD-11095**	Green	Dark Green	Ovate	Light Green	Round	Brown	Light Green	Indeterminate	Green	Short beak	Straight	Slightly curved	White	White	White	White
28	**BD-11097**	Green	Light Green	Ovate	Light Green	Flat elongate	Black	Light Green	Indeterminate	Light green	Short beak	Straight	Slightly curved	Light purple	Light purple	Light purple	Light violet
29	**BD-11098**	Green	Light Green	Ovate	Light Green	Round	Dark-brown	Light Green	Indeterminate	Light green	Short beak	Straight	Slightly curved	White	White	White	White
30	**BD-11099**	Green	Light Green	Ovate	Light Green	Round	Black	Light Green	Indeterminate	Light green	Short beak	Straight	Slightly curved	Purple	Purple	Purple	Light purple

Table S1. (Continued)																	
**Serial no.**	**Name of the germplams**	**Cotyledon colour (CC)**	**Leaf colour (LC)**	**Leaf shape (LS)**	**Vein colour (VC)**	**Seed shape (SS)**	**Seed colur (SeC)**	**Stem colour (StC)**	**Growth habit (GH)**	**Pod colour (PC)**	**Pod beak (PB)**	**Pod constription (PCo)**	**Pod curvature (PCu)**	**Standard petal colour (SPC)**	**Wing petal colour (WPC)**	**Keel petal colour (KPC)**	**Flower colour (FC)**
	**Commercially released and locally collected Lablab purpureus varieties**																
31	**BARI Sheem-1**	Green	Light Green	Ovate	Light Green	Round	Black	Light Green	Indeterminate	Dark green	Short beak	Straight	Moderately curved	Purple	Purple	Purple	Pink
32	**BARI Sheem-2**	Green	Dark Green	Ovate	Dark Green	Oval elongate	Brown	Light Green	Indeterminate	Dark green	Short beak	Straight	Straight	White	White	White	White
33	**BARI Sheem-3**	Green	Dark Green	Ovate	Dark Green	Flat oval	Brown	Light Green	Indeterminate	Green	Short beak	Straight	Slightly curved	White	White	White	White
34	**BARI Sheem-4**	Green	Dark Green	Ovate	Dark Green	Elongate	Black	Light Green	Indeterminate	Light green	Long beak	Straight	Moderately curved	White	White	White	White
35	**BARI Sheem-5**	Light Green	Dark Green	Ovate	Dark Green	Round	Brown	Light Green	Determinate	Light green	Long beak	Straight	Slightly curved	White	White	White	White
36	**BARI Sheem-6**	Light Green	Dark Green	Ovate	Purple	Oval	Black	Purple	Indeterminate	Light green	Long beak	Straight	Slightly curved	Purple	Purple	Purple	Pink
37	**BARI Sheem-7**	Green	Dark Green	Ovate	Dark Green	Oval	Brown	Light Green	Indeterminate	Dark green	Long beak	Straight	Straight	White	White	White	White
38	**BARI Sheem-8**	Green	Light Green	Ovate	Light Green	Elongate oval	Dark-brown	Green	Indeterminate	Light green	Short beak	Slightly constricted	Moderately curved	Cream	Cream	Cream	White
39	**IPSA Sheem-2**	Green	Dark Green	Ovate	Dark Green	Round	Brown	Light Green	Indeterminate	Light green & dark purple border	Short beak	Straight	Moderately curved	White	White	White	White
40	**Khisamoti**	Light Green	Dark Green	Ovate	Dark Green	Elongate oval	Brown	Purple	Indeterminate	Light green	Short beak	Light constriction	Slightly curved	White	White	White	Pink

Table S1. (Continued)

Serial no.	Name of the germplams	Cotyledon colour (CC)	Leaf colour (LC)	Leaf shape (LS)	Vein colour (VC)	Seed shape (SS)	Seed colur (SeC)	Stem colour (StC)	Growth habit (GH)	Pod colour (PC)	Pod beak (PB)	Pod constription (PCo)	Pod curvature (PCu)	Standard petal colour (SPC)	Wing petal colour (WPC)	Keel petal colour (KPC)	Flower colour (FC)
41	**Rifa**	Green	Light Green	Ovate	Light Green	Flat oval	White creamy	Green	Indeterminate	Light green	Short beak	Straight	Slightly curved	White	White	White	White
42	**Goal Goda**	Light Green	Light Green	Ovate	Light Green	Flat oval	Dark-brown	Purple	Indeterminate	Dark green	Short beak	Straight	Slightly curved	Light purple	Light purple	Light purple	Pink
43	**Nodi**	Light Green	Light Green	Ovate	Light Green	Flat oval	Dark-brown	Purple	Indeterminate	Dark green	Short beak	Straight	Slightly curved	Light purple	Light purple	Light purple	Pink
44	**Noldoc**	Light Green	Light Green	Ovate	Light Green	Flat oval	Dark-brown	Light purple	Indeterminate	Green	Short beak	Straight	Slightly curved	Purple	Purple	Purple	Pink
45	**Ali**	Green	Dark Green	Ovate	Light Green	Oval	Black	Green	Indeterminate	Light green	Short beak	Straight	Slightly curved	Light purple	Light purple	Light purple	Pink
46	**Laluri**	Green	Dark Green	Ovate	Dark Green	Round	Brown	Light Green	Indeterminate	Light green	Long beak	Straight	Slightly curved	White	White	White	White
47	**Mostofa**	Light Green	Dark Green	Ovate	Dark Green	Round	Black	Purple	Indeterminate	Light green	Long beak	Straight	Slightly curved	Light purple	Light purple	Light purple	Pink
48	**Kaloputi**	Light Green	Dark Green	Ovate	Dark Green	Round	Black	Purple	Indeterminate	Dark green	Short beak	Slightly constricted	Slightly curved	Light purple	Light purple	Light purple	Pink
49	**Chonchol**	Green	Dark Green	Ovate	Dark Green	Round	Black	Green	Indeterminate	Light green	Short beak	Straight	Slightly curved	White	White	White	Pink
50	**BARI Sheem-9**	Green	Light Green	Ovate	Light Green	Oval	Brown	Light Green	Indeterminate	Green	Short beak	Straight	Slightly curved	White	White	Light yellow	White

Table S2. The conversion value of the sixteen qualitative morphological traits of 50 *Lablab purpureus* germplasms

Serial no.	Germplasm	Cotyledon colour (CC)	Leaf colour (LC)	Leaf shape (LS)	Vein colour (VC)	Seed shape (SS)	Seed colur (SeC)	Stem colour (StC)	Growth habit (GH)	Pod colour (PC)	Pod beak (PB)	Pod constriptio n (PCo)	Pod curvature (PCu)	Standard petal colour (SPC)	Wing petal colour (WPC)	Keel petal colour (KPC)	Flower colour (FC)
1	BD-10798	2	3	1	5	3	2	5	1	13	3	3	3	6	6	6	6
2	BD-10799	1	4	2	3	1	3	3	1	5	3	3	2	2	2	2	3
3	BD-10800	1	4	2	3	1	3	1	1	5	1	3	1	3	3	3	3
4	BD-10801	1	4	2	5	1	3	4	1	4	3	3	2	3	3	3	2
5	BD-10802	1	4	2	5	1	3	1	1	13	1	2	1	3	3	3	2
6	BD-10803	1	4	2	5	3	4	1	1	13	1	5	2	6	6	6	6
7	BD-10804	2	4	2	3	3	3	4	1	3	3	3	2	2	2	2	2
8	BD-10805	1	4	2	3	4	3	4	1	8	1	5	2	5	5	5	3
9	BD-10806	1	4	2	3	3	3	1	1	10	1	5	2	6	6	6	6
10	BD-10807	1	4	2	3	1	3	4	1	5	3	5	2	3	3	2	3
11	BD-10808	1	4	2	4	3	3	3	1	11	3	5	2	3	3	3	7
12	BD-10809	1	1	2	3	1	3	3	1	13	3	5	2	2	2	2	3
13	BD-10811	1	4	2	3	3	3	2	1	13	3	3	1	5	5	5	7
14	BD-10812	1	4	2	4	3	3	3	1	9	1	5	2	3	3	3	3
15	BD-10813	1	4	2	5	3	3	2	1	13	3	3	1	3	3	3	2
16	BD-10814	1	4	2	5	3	2	1	1	12	3	5	2	6	6	7	6
17	BD-10815	1	1	2	4	1	3	2	1	13	3	3	2	3	3	2	2
18	BD-10816	1	4	2	4	1	3	3	1	2	3	5	2	3	3	3	2
19	BD-10818	1	4	2	5	4	3	2	1	12	3	5	2	3	3	2	7
20	BD-11087	2	1	2	1	1	3	1	1	2	3	4	1	3	3	3	7
21	BD-11088	2	1	2	1	6	2	1	1	12	3	5	2	6	6	7	6
22	BD-11089	1	1	2	3	1	3	4	1	1	3	5	1	2	2	2	2
23	BD-11090	1	2	2	1	4	3	2	1	13	3	5	1	3	3	7	2
24	BD-11091	1	1	2	4	6	3	4	1	1	3	5	1	1	1	4	7
25	BD-11092	1	2	2	3	6	2	2	1	2	2	1	2	2	2	2	8
26	BD-11093	2	1	2	4	1	3	4	1	3	1	5	1	3	3	3	7
27	BD-11095	2	1	2	1	4	2	1	1	13	3	5	2	6	6	6	6
28	BD-11097	2	2	2	1	6	3	1	1	12	3	5	2	2	2	2	7
29	BD-11098	2	2	2	1	4	1	1	1	12	3	5	2	6	6	6	6
30	BD-11099	2	2	2	1	4	3	1	1	12	3	5	2	3	3	3	2
31	BARI Sheem-1	2	2	2	1	4	3	1	1	2	3	5	1	3	3	3	5
32	BARI Sheem-2	2	1	2	2	2	2	1	1	2	3	5	3	6	6	6	6
33	BARI Sheem-3	2	1	2	2	1	2	1	1	13	3	5	2	6	6	6	6
34	BARI Sheem-4	2	1	2	2	5	3	1	1	12	1	5	1	6	6	6	6
35	BARI Sheem-5	1	1	2	2	4	2	1	2	12	1	5	2	6	6	6	6
36	BARI Sheem-6	1	1	2	4	3	1	4	1	12	1	5	2	3	3	3	5
37	BARI Sheem-7	2	1	2	2	3	2	1	1	2	1	5	3	6	6	6	6
38	BARI Sheem-8	2	2	2	1	7	1	5	1	12	3	3	1	4	4	4	6
39	IPSA Sheem-2	2	1	2	2	4	2	1	1	6	3	5	1	6	6	6	6
40	Khisamoti	1	1	2	2	7	2	4	1	12	3	1	2	6	6	6	5
41	Rifa	2	2	2	1	1	4	5	1	12	3	5	2	6	6	6	6
42	Goal Goda	1	2	2	1	1	1	4	1	2	3	5	2	2	2	2	5
43	Nodi	1	2	2	1	1	3	4	1	2	3	5	2	2	2	2	5
44	Noldoc	1	2	2	1	1	1	3	1	13	3	5	2	3	3	3	5
45	Ali	2	1	2	1	3	1	5	1	12	3	5	2	2	2	2	5
46	Laluri	2	1	2	2	4	2	1	1	12	1	5	2	6	6	6	6
47	Mostofa	1	1	2	2	4	3	4	1	12	1	5	2	2	2	2	5
48	Kaloputi	1	1	2	2	4	3	4	1	2	3	3	2	2	2	2	5
49	Chonchol	2	1	2	2	4	3	5	1	12	3	5	2	6	6	6	5
50	BARI Sheem-9	2	2	2	1	3	2	1	1	13	3	5	2	6	6	7	6
		1: Light Green, 2: Green	1: Light Green, 2: Dark Green, 3: Pale Green, 4: Green	1: Ovate lanceolate, 2: Ovate	1: Light Green, 2: Dark Green, 3: Light Purple, 4: Purple,5: Green	1: Flat Oval, 2:Oval elongate, 3: Oval, 4: Round, 5: Elongate, 6: Flat Elongate, 7: Elongate Oval	1: Dark Brown, 2: Brown, 3: Black, 4: White Creamy	1: Light Green, 2: Very light purple, 3: Light Purple, 4: Purple, 5:Green,	1:Indeterminate, 2: Determinate	1: Dark green, 2: Dark green with purple, 3: Green with light purple border, 4: Green with slightly purple, 5: Green with purple, 6: Light green & dark purple border, 7: Light green with purple border, 8: Light purple with purple border, 9: Light purple with whitish green, 10: light whitish green, 11: lihgt purple, 12: light green, 13: Green/ green	1: long beak, 2: very short beak, 3: short beak	1: light constriction, 2: moderately constricted, 3: slightly constricted, 4: straight, flat, 5: staigh	1: Moderately curved, 2: Slightly curved, 3: Straight	1: Very Light purple, 2: Light purple, 3: purple, 4: cream, 5: pink, 6: white	1: Very Light purple, 2: Light purple, 3: purple, 4: cream, 5: pink, 6: white	1: Very Light purple, 2: Light purple, 3: purple, 4: cream, 5: pink, 6: white	1: Very Light purple, 2: Light purple, 3: purple, 4: cream, 5: pink, 6: white

Table S3. Clustering of the 50 evaluated country bean germplasm based on qualitative and quantitative morphological traits as per agglomerative hierarchical clustering (AHC) analysis

Clustering based on qualitative morphological traits			Clustering based on quantitative morphological traits*			
Number of germplasms in each cluster			Number of germplasms in each cluster			
**Cluster I**	**Cluster II**	**Cluster III**	**Cluster I**	**Cluster II**	**Cluster III**	**Cluster IV**
15	17	18	13	7	29	1
Ali	BARI Sheem-9	BARI Sheem-1	BD-10808	Noldoc	BD-10802	Rifa
BARI Sheem-6	BARI Sheem-3	BARI Sheem-2	BD-10809	Ali	BD-10805	
BD-10802	BARI Sheem-4	BARI Sheem-7	BD-10812	BARI Sheem-6	BD-10813	
BD-10805	BARI Sheem-5	BD-10799	BD-10815	Chonchol	BD-10818	
BD-10808	BARI Sheem-8	BD-10800	BD-10798	BD-10800	BD-11090	
BD-10809	BD-10798	BD-10801	BD-10811	BD-11092	BD-11097	
BD-10812	BD-10803	BD-10804	BARI Sheem-5	Nodi	BD-11099	
BD-10813	BD-10806	BD-10807	Goal Goda		Mostofa	
BD-10815	BD-10811	BD-10816	BD-10799		BD-10803	
BD-10818	BD-10814	BD-11087	BD-10801		BD-10806	
BD-11090	BD-11088	BD-11089	BD-10807		BD-10814	
BD-11097	BD-11095	BD-11091	BD-10816		BD-11088	
BD-11099	BD-11098	BD-11092	BD-11087		BD-11095	
Mostofa	Chonchol	BD-11093			BD-11098	
Noldoc	Khisamoti	Goal Goda			BARI Sheem-3	
	Laluri	IPSA Sheem-2			BARI Sheem-4	
	Rifa	Kaloputi			BARI Sheem-8	
		Nodi			BARI Sheem-9	
					Khisamoti	
					Laluri	
					BD-10804	
					BD-11089	
					BD-11091	
					BD-11093	
					BARI Sheem-1	
					BARI Sheem-2	
					BARI Sheem-7	
					IPSA Sheem-2	
					Kaloputi	

Yellow, blue and light orange color in the quantitative clusters represents the distribution of cluster I, II and III germplasms based on qualitative traits from left side

Table S4. Mean of seventeen quantitative morphological traits of *Lablab* germplasm from Bangladesh

Serial no.	Name of the germplasm	SW	SV	SL	SeW	SLA	LL	LW	LeL	PH	APW	PL	PW	PT	NFPS	SpL	DFF	TNSPP
1	BD-10798	4.30	4.00	19	11	10.52	10.00	8.50	16.00	245.31	6.70	11.50	2.60	0.96	8.0	10.0	60.0	13.0
2	BD-10799	3.46	3.00	12	8	12.17	11.50	10.00	29.00	250.19	14.20	13.00	4.00	0.46	9.0	26.0	65.0	20.0
3	BD-10800	2.77	3.00	13	8	11.80	11.50	8.90	23.00	258.90	7.90	14.00	2.50	0.90	10.0	15.5	86.0	18.0
4	BD-10801	4.21	4.00	15	10	9.48	9.30	7.00	29.50	259.08	11.70	11.50	3.00	0.60	18.0	29.0	63.0	13.0
5	BD-10802	3.65	4.00	13	8	11.66	11.50	8.50	28.50	251.88	7.20	11.00	2.00	0.50	20.0	21.0	86.0	30.0
6	BD-10803	5.84	5.00	12	8	11.62	11.00	9.50	21.50	250.19	7.20	8.50	2.70	1.10	21.0	33.0	90.0	17.0
7	BD-10804	4.92	5.00	9	11	13.73	11.80	13.90	39.00	260.35	32.20	15.90	3.50	0.60	22.0	41.0	90.0	13.0
8	BD-10805	4.73	5.00	9	10	14.04	13.50	11.00	28.00	251.46	4.70	9.50	2.50	0.70	20.0	28.0	86.0	40.0
9	BD-10806	4.27	4.00	9	8	11.96	11.00	10.50	37.00	247.23	11.30	16.00	1.20	0.80	16.0	32.0	87.0	8.0
10	BD-10807	3.80	3.50	14	9	11.48	10.50	10.20	27.00	250.19	29.00	15.00	4.20	0.50	17.0	32.0	57.0	10.0
11	BD-10808	6.98	5.50	15	11	10.90	10.50	8.50	26.50	245.53	18.30	7.50	2.50	0.90	11.0	24.0	62.0	18.0
12	BD-10809	4.11	3.00	13	10	12.34	11.50	10.50	27.50	271.78	6.00	11.50	2.60	0.60	16.0	32.0	62.0	19.0
13	BD-10811	4.25	4.00	12	9	14.04	13.50	11.00	28.00	259.08	5.80	8.50	2.30	0.60	22.0	36.0	67.0	22.0
14	BD-10812	3.72	3.00	9	7	14.14	13.50	11.30	31.50	254.84	6.00	8.50	2.30	1.00	17.0	27.0	66.0	30.0
15	BD-10813	5.25	4.00	13	9	10.69	10.00	9.00	23.00	265.43	8.50	7.50	2.50	0.93	18.0	32.0	91.0	15.0
16	BD-10814	4.69	4.00	13	9	11.24	10.50	9.50	25.50	254.00	8.00	8.50	2.30	0.40	16.0	28.0	86.0	17.0
17	BD-10815	4.24	3.00	12	9	10.69	10.00	9.00	25.50	261.62	17.00	13.00	4.50	0.60	20.0	30.0	64.0	13.0
18	BD-10816	4.01	3.50	10	9	12.17	11.50	10.00	28.50	261.62	12.50	12.50	4.30	0.53	18.0	29.0	64.0	21.0
19	BD-10818	4.23	3.00	10	10	11.07	10.50	9.00	25.00	256.54	8.50	7.50	2.50	0.73	16.0	31.0	86.0	14.0
20	BD-11087	3.03	4.00	11	9	11.14	10.50	9.20	22.50	265.85	11.00	8.50	3.00	0.63	16.0	28.0	60.0	9.0
21	BD-11088	4.70	4.00	15	9	14.25	14.00	10.50	33.00	256.54	8.50	9.00	2.50	0.66	22.0	26.0	86.0	26.0
22	BD-11089	4.55	4.00	12	8	12.24	11.50	10.20	30.00	245.53	16.50	15.00	4.00	0.56	16.0	28.0	87.0	11.0
23	BD-11090	3.00	3.00	10	8	10.73	10.50	8.00	20.50	279.40	5.70	8.50	2.70	0.83	18.0	28.0	86.0	15.0
24	BD-11091	3.66	4.00	11	7	10.14	9.50	8.50	20.50	266.70	19.50	14.00	3.50	0.56	20.0	36.0	86.0	8.0
25	BD-11092	3.39	4.00	18	7	12.39	12.00	9.50	23.00	260.77	11.00	10.50	1.60	0.63	10.0	16.0	87.0	10.0
26	BD-11093	6.15	6.00	19	11	11.66	10.60	10.50	28.00	242.57	9.50	10.00	2.50	0.80	17.0	32.0	86.0	32.0

27	BD-11095	4.93	4.00	9	9	9.45	9.00	7.60	22.50	259.08	9.50	10.20	2.30	1.00	16.0	23.0	80.0	14.0
28	BD-11097	3.32	4.00	8	8	8.51	7.50	8.20	20.50	256.54	9.50	11.00	2.50	0.56	18.0	29.0	86.0	18.0
29	BD-11098	4.48	4.50	8	7	10.21	10.00	7.60	22.50	262.89	6.50	8.50	2.00	0.91	23.0	30.0	82.0	24.0
30	BD-11099	3.17	2.50	10	8	8.61	7.50	8.50	17.50	253.15	7.20	11.00	2.00	0.66	22.0	41.0	86.0	24.0
31	BARI Sheem-1	3.77	4.00	14	9	7.55	7.00	6.50	19.00	246.38	12.50	11.00	3.00	0.70	30.0	35.2	86.0	22.0
32	BARI Sheem-2	3.65	4.50	15	8	10.18	10.00	7.50	22.50	238.76	12.00	11.00	2.50	0.80	26.0	30.0	86.0	20.0
33	BARI Sheem-3	5.48	6.00	15	10	12.34	11.50	10.50	23.00	244.69	11.50	9.30	3.20	0.70	32.0	60.0	82.0	23.0
34	BARI Sheem-4	5.42	4.50	15	10	12.34	11.50	10.50	26.00	247.65	12.00	14.50	2.00	0.90	26.0	36.5	86.0	25.0
35	BARI Sheem-5	3.23	3.00	10	12	10.73	10.50	8.00	27.50	264.16	8.00	12.00	2.50	0.60	26.0	41.1	45.0	18.0
36	BARI Sheem-6	3.76	4.00	15	10	12.43	12.50	8.50	22.50	246.38	12.00	14.50	2.00	1.00	22.0	7.8	87.0	14.0
37	BARI Sheem-7	3.08	4.00	10	9	11.17	10.50	9.30	21.00	260.35	8.50	12.00	3.00	0.70	22.0	43.0	80.0	15.0
38	BARI Sheem-8	5.09	4.00	15	10	12.22	12.00	9.00	23.00	259.08	12.00	14.00	2.00	0.80	28.0	44.7	82.0	19.0
39	IPSA Sheem-2	3.64	3.50	12	10	11.17	10.50	9.30	21.00	264.16	6.50	7.50	2.00	0.70	36.0	31.3	87.0	29.0
40	Khisamoti	3.17	3.00	14	12	11.14	10.50	9.20	21.50	257.39	5.70	8.50	2.70	0.60	24.0	39.4	91.0	11.0
41	Rifa	1.99	1.99	12	10	10.52	10.00	8.50	27.50	60.96	4.00	8.80	3.00	0.60	18.0	36.5	70.0	13.0
42	Goal Goda	4.88	4.88	14	10	12.13	13.00	6.50	33.00	238.76	22.90	16.00	2.50	1.10	14.0	8.1	62.0	9.0
43	Nodi	4.00	4.00	12	11	13.23	14.00	7.50	32.00	279.40	11.80	17.90	3.20	0.70	13.0	9.9	80.0	9.0
44	Noldoc	3.95	3.95	13	10	12.73	12.00	10.50	27.70	248.92	10.90	22.90	2.00	0.90	9.0	7.2	76.0	6.0
45	Ali	3.45	3.45	19	9	13.03	12.40	10.50	20.00	253.15	12.00	8.90	2.50	0.70	8.0	23.3	89.0	6.0
46	Laluri	5.35	5.35	12	8	10.96	10.80	8.00	28.50	243.84	9.70	8.70	2.00	0.80	32.0	49.8	80.0	29.0
47	Mostofa	5.88	5.88	14	11	13.57	14.00	8.50	27.00	259.08	9.90	8.70	3.00	0.80	37.0	58.4	88.0	29.0
48	Kaloputi	5.45	5.45	12	10	13.08	13.00	9.30	30.00	235.37	7.90	9.70	2.00	0.70	21.0	31.2	76.0	17.0
49	Chonchol	5.55	5.55	16	10	11.45	11.00	9.00	25.50	251.46	8.80	11.80	2.00	0.70	7.0	8.6	80.0	5.0
50	BARI Sheem-9	6.80	6.80	15	11	12.62	12.00	10.20	31.00	256.54	12.70	9.90	2.70	0.72	36.0	42.0	86.0	19.0

The 17 quantitative morphological traits are 10-seed weight (SW) (g), 10-seed volume (SV) (cm^3), seed length (SL) (mm), seed width (SeW) (mm), single leaflet area (SLA) (cm^2), leaflet length (LL) (cm), leaflet width (LW) (cm), leaf length (LeL) (cm), plant height (PH) (cm), average pod weight (APG) (g), pod length (PL) (cm), pod width (PW) (cm), pod thickness (PT) (cm), no. of flower per spike (NFPS), spike length (SpL) (cm), days to first flowering (DFF), and total no. of spike per plant (TNSPP
